# RNAi library screening reveals Gβ1, Casein Kinase 2 and ICAP‐1 as novel regulators of LFA‐1‐mediated T cell polarity and migration

**DOI:** 10.1111/imcb.12838

**Published:** 2024-11-28

**Authors:** Antje Haap‐Hoff, Michael Freeley, Eugene Dempsey, Dara Dunican, Emily Bennett, Denise Triglia, Joanna Skubis‐Zegadlo, Anthony Mitchell Davies, Dermot Kelleher, Aideen Long

**Affiliations:** ^1^ Trinity Translational Medicine Institute & Department of Clinical Medicine, Trinity College Dublin St James's Hospital Dublin Republic of Ireland; ^2^ Present address: School of Biotechnology Dublin City University Dublin Ireland; ^3^ Present address: Faculty of Medicine University of British Columbia Vancouver BC Canada

**Keywords:** cell migration, cell polarity, T cells

## Abstract

The α_L_β_2_ integrin LFA‐1 plays a key role in T‐cell adhesion to the endothelial vasculature and migration into both secondary lymphoid organs and peripheral tissues via interactions with its target protein ICAM‐1, but the pathways that regulate LFA‐1‐mediated T‐cell polarity and migration are not fully understood. In this study we screened two RNAi libraries targeting G protein‐coupled receptors (GPCR)/GPCR‐associated proteins and kinases in a HuT 78 T cell line model of LFA‐1‐stimulated T‐cell migration. Based on staining of the actin cytoskeleton, multiple parameters to measure cell morphology were used to assess the contribution of 1109 genes to LFA‐1‐mediated T‐cell polarity and migration. These RNAi screens identified a number of both novel and previously identified genes that either increased or decreased the polarity and migratory capacity of these cells. Following multiparametric analysis, hierarchical clustering and pathway analysis, three of these genes were characterized in further detail using primary human T cells, revealing novel roles for the heterotrimeric G protein subunit Gβ1 and Casein Kinase 2 in LFA‐1‐mediated T‐cell polarity and migration *in vitro*. Our studies also highlighted a new role for ICAP‐1, an adaptor protein previously described to be associated with β1 integrins, in β2 integrin LFA‐1‐directed migration in T cells. Knockdown of ICAP‐1 expression in primary T cells revealed a role in cell polarity, cell velocity and transmigration towards SDF‐1 for this adaptor protein. This study therefore uncovers new roles for GPCR/GPCR‐associated proteins and kinases in T‐cell migration and provides potential novel targets for modulation of the T‐cell immune response.

## Graphical abstract

In this study, we screened RNAi libraries, targeting kinases and G protein‐coupled receptors/G protein‐coupled receptor associated proteins for regulators of T‐cell polarity and migration. We identified that Gβ1, casein kinase 2 and ICAP‐1 play key roles in T‐cell adhesion, polarity and migration on ICAM‐1.

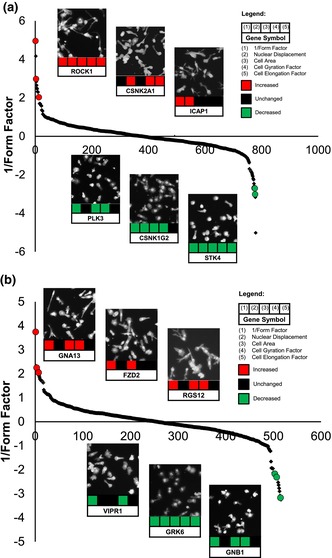

## INTRODUCTION

The migration of T cells into secondary lymphoid organs and their recruitment to inflamed peripheral tissues is crucial for immune responses to pathogens and microorganisms.^1^ Unregulated T‐cell activation and migration, however, is also a major contributing factor in many autoimmune diseases and inflammatory disorders such as multiple sclerosis and inflammatory bowel disease.^2^ T‐cell migration is guided by multistep interactions between receptors expressed on T cells and their counter‐ligands expressed on endothelial cells lining blood vessels at lymph node entry points (known as high endothelial venules) and peripheral tissues. For example, it is well established that selectins, G‐protein‐coupled chemokine receptors and integrins expressed on T cells play important roles in the context of migration by facilitating rolling, firm adhesion and transmigration (diapedesis) into tissues.^1^ Firm adhesion is mediated via signaling through G‐protein‐coupled chemokine receptors, which detect endothelial‐bound chemokines and initiate intracellular signaling cascades that increase the adhesive capacity of integrins for their ligands on the vascular wall by a process known as “inside‐out” activation.^3^, ^4^ Ligand‐bound integrins subsequently initiate T‐cell polarization and migration, resulting in T‐cell crawling along the blood vessel wall in search of a suitable entry site into the tissue. T‐cell polarization and migration are regulated via dynamic reorganization of the actin and microtubule cytoskeleton, which provide the protrusive and contractile forces necessary for cellular locomotion.^4^ Migrating T cells typically adopt a morphology that resembles a “hand mirror”, with two distinct compartments known as the leading edge (where filamentous actin and chemokine receptors are localized) and the trailing tail/uropod.^5^

The α_L_β_2_ integrin (also known as Lymphocyte Function‐Associated Antigen‐1; LFA‐1) plays a crucial role in the adhesion, cytoskeletal reorganization and motility of T cells.^4^ LFA‐1 is maintained on resting T cells in a bent conformation with low affinity for its ligand ICAM‐1 expressed on endothelial cells, but adopts at least two extended conformations following chemokine‐induced inside‐out activation that increase its affinity for ICAM‐1 to mediate firm adhesion.^3^, ^4^ A number of intracellular proteins are known to regulate inside‐out activation of LFA‐1 in T cells. These include the adaptor proteins talin‐1 and kindlin‐3, which directly modulate LFA‐1 activation by binding to distinct motifs located on the β chain of the integrin.^3^, ^4^ Although the intracellular signaling cascades that couple ligand‐bound integrins to the cytoskeleton to direct T‐cell migration are less well understood, we and others have demonstrated key roles for PKCβ (a serine/threonine kinase),^6^ ZAP‐70,^7^, ^8^ myosin IIA^9^, ^10^ and the scaffolding protein Rack1^11^ in LFA‐1‐mediated migration and cytoskeletal reorganization. Nonetheless, many factors controlling this process have yet to be identified.

RNA interference (RNAi) is a powerful tool for silencing of gene expression, as it enables loss‐of‐function studies to be carried out in a highly specific manner.^12^ Furthermore, screening of RNAi libraries has made it possible to identify genes involved in any cellular process in an unbiased manner.^13^ This has led to the discovery of novel genes regulating a host of cellular processes in both non‐vertebrate and vertebrate cells, including genes regulating cell cycle, migration, viability, endocytosis and host–pathogen interactions.^14^, ^15^, ^16^ When coupled with technologies such as image‐based high content analysis (HCA), RNAi library screening is ideal for analyzing complex cellular phenotypes, as the automated image acquisition permits large cell populations to be rapidly analyzed (an important point when one considers that gene silencing may not be uniform and/or completely suppressed in the entire cell population).^17^ Furthermore, tens to thousands of descriptive features/parameters can potentially be extracted from each cell, enabling high‐dimensional analysis of cellular phenotypes increasing the likelihood of detecting novel gene functions.^18^, ^19^

In this study, we combined RNAi library screening with an image‐based HCA platform to identify novel genes regulating LFA‐1‐induced T‐cell polarity and migration. We screened two RNAi libraries targeting kinases or GPCR/GPCR‐associated proteins, which were selected based on roles for several of these family members in T‐cell polarity and migration,^4^ and thereby assessed the contribution of 1109 genes to LFA‐1‐mediated T‐cell polarity and migration. The HuT 78 T cell line stimulated with an immobilized antibody specific for the αL chain of the LFA‐1 integrin was used as a model of T cell polarization and migration.^6^ The cells were then fixed and stained for the actin cytoskeleton to analyze changes in cell morphology using multiple parameters by high‐content image analysis. A selection of siRNAs that affected LFA‐1‐induced T‐cell polarization in the HuT‐78 T cell line were studied in more detail in primary human T cells stimulated with ICAM‐1 (the natural ligand for the LFA‐1 integrin). Using this approach, we identified the heterotrimeric G protein subunit Gβ1, the adaptor protein ICAP‐1 and Casein Kinase 2 as novel regulators of LFA‐1‐ mediated T‐cell polarity and migration *in vitro*. These studies add weight to the validity of using RNAi screens to discover new candidates involved in cell migration and for the identification of novel targets for the treatment of T cell‐mediated inflammatory diseases.

## RESULTS

### Identification of novel genes regulating T‐cell migration by RNAi library screening

**Figure 1:**
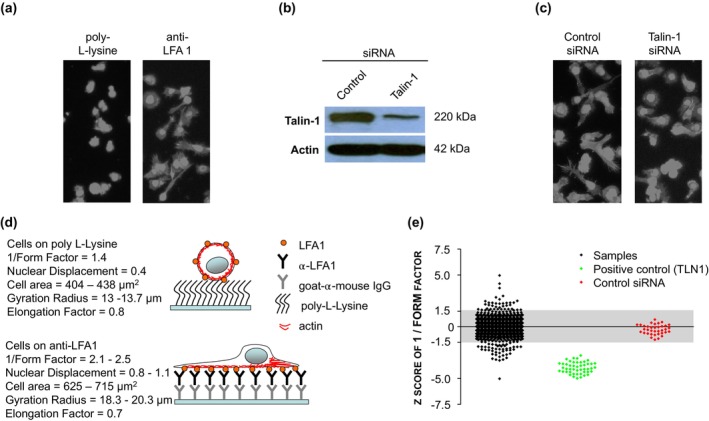
Summary of RNAi library screening approach in HuT 78 T cells to identify novel regulators of LFA‐1‐mediated T cell polarity and migration. **(a)** Incubation of HuT 78 T cells on poly L‐lysine or immobilized anti‐LFA‐1 antibodies induces a non‐polarized or polarized morphology, respectively. The cells were fixed and stained with phalloidin‐TRITC and Hoechst 33258 and imaged by high content microscopy under 10× magnification. **(b)** Transfection of siRNAs targeting talin‐1 into HuT 78 T cells by electroporation induces silencing of Talin‐1 expression levels as confirmed by Western blotting using talin‐1 antibodies. Actin served as a loading control. **(c)** Silencing talin‐1 expression in HuT 78 T cells perturbs T‐cell morphology on anti‐LFA‐1 antibodies. Cells were fixed and stained with phalloidin‐TRITC and Hoechst 33258 and imaged by high content microscopy under 10× magnification. **(d)** Typical cell morphology parameters following incubation on poly L‐lysine or immobilized anti‐LFA‐1 antibodies and schematic of anti‐LFA‐1 migration assay and F‐Actin localization. **(e)** Robust *Z*‐scores of 1/Form factor cell morphology parameter from a single RNAi screen in HuT 78 T cells.

Stimulation of T cells through the LFA‐1 integrin with the physiological ligand ICAM‐1, or with an antibody specific for the α_L_ chain of LFA‐1, induces cellular polarization and the acquisition of a migratory phenotype^6^ (Figure 1a). We used the anti‐LFA‐1 migration assay to screen two RNAi libraries for novel genes involved in LFA‐1‐mediated polarity and migration in the HuT 78 T cell line. Because the cross‐linking stimulus triggers T‐cell polarization and migration independently of chemokine‐induced “inside‐out” integrin activation, we assume that the acquisition of a migratory phenotype in this context mimics post‐integrin activation and thus “outside‐in” integrin signaling. The RNAi libraries targeting human kinases (719 genes) and human GPCR/GPCR‐associated proteins (390 genes) were provided in multiple 96‐well plates as siGenome SMARTpools, such that individual wells of the plates contained four distinct siRNAs targeting the expression of a single gene. Included on every plate were non‐targeting control SMARTpool siRNAs. Because T cells are well‐known as “hard‐to‐transfect”,^20^ electroporation was used to deliver the siRNAs into the cells using 96‐well electroporation plates. Electroporation conditions for the RNAi screens were optimized with siRNAs targeting talin‐1 and other proteins. Delivering 500 nM of talin‐1‐specific siRNAs to the HuT 78 T cells in three consecutive pulses resulted in a knockdown efficiency of > 80% after 72 h in comparison with cells electroporated with control non‐targeting siRNAs, as demonstrated by Western blotting (Figure 1b). While talin‐1 is known to mediate “inside‐out” activation of LFA‐1 in response to chemokine stimulation, we also found that silencing talin‐1 expression in HuT 78 T cells inhibited their ability to polarize efficiently on anti‐LFA‐1 (Figure 1c). This is consistent with reports in other cell types that have demonstrated additional roles for talin‐1 in coupling ligand‐bound integrins to the cytoskeleton and hence roles in “outside‐in” signaling.^21^, ^22^ siRNAs targeting the expression of talin‐1 were therefore used in the RNAi library screens as positive controls to inhibit the anti‐LFA‐1‐mediated migratory phenotype. An outline of the RNAi screening procedure and analysis of T‐cell morphology/migration is as follows: after electroporating the HuT 78 T cells with the RNAi libraries and then incubating the cells for 72 h to enable silencing of gene expression, the cells were subsequently incubated in 96‐well plates coated with anti‐LFA‐1 antibodies to allow the cells to develop a polarized morphology. The cells were then fixed and stained with phalloidin‐TRITC and Hoechst 33258 to label F‐actin and the nucleus, respectively. An image‐based HCA platform and multiparametric analysis was used to quantify the morphology of the cells and was based on F‐actin staining. The parameters 1/(Form factor), nuclear displacement, cell area, gyration radius and elongation factor were chosen as read‐outs of migration‐related morphological changes.^19^ Typical values for these parameters in resting and migrating T cells are shown in Figure 1d. The 1/(Form factor) parameter is a roundness index, with values ranging from 1 to infinity, where 1 is a perfect circle. 1/(Form factor) increases as T cells adopt a migratory phenotype and is thus a useful measure of T‐cell migration. The nuclear displacement parameter describes the position of the nucleus relative to the center of the cell body. In non‐migrating T cells, the nucleus is usually positioned in the center of the cell and therefore has a low nuclear displacement value. In migrating T cells, nuclear displacement values increase because the nucleus translocates towards the leading edge of the cell and away from the center of the cell body. The gyration radius is a measure of the spread of the cell, while the elongation factor measures the ratio of the length of the cell to the width of the cell. Both of these parameters, as well as the cell area, increase as T cells polarize on anti‐LFA‐1 (Figure 1d). We used the *Z*‐scoring system to define the effects of siRNAs on these morphology parameters (see Methods). The effects of siRNAs were defined as being statistically significant if they exceeded a *Z*‐score threshold of ±1.5. Figure 1e illustrates the *Z*‐score distribution for the morphology parameter 1/(Form factor) for all siRNAs from a representative RNAi screen. All of the siRNAs targeting talin‐1 in the screen significantly decreased the 1/(Form factor) parameter as expected (with *Z* scores ranging between −1.5 and −5.0), whereas the control non‐targeting siRNAs had *Z* scores between −1.5 and +1.5 for this parameter and thus were not significant. Other siRNAs from the representative RNAi screen affected the 1/(Form factor) parameter to varying extents, as these siRNAs either significantly decreased this parameter, significantly increased this parameter or had no effect (Figure 1e).

**Figure 2:**
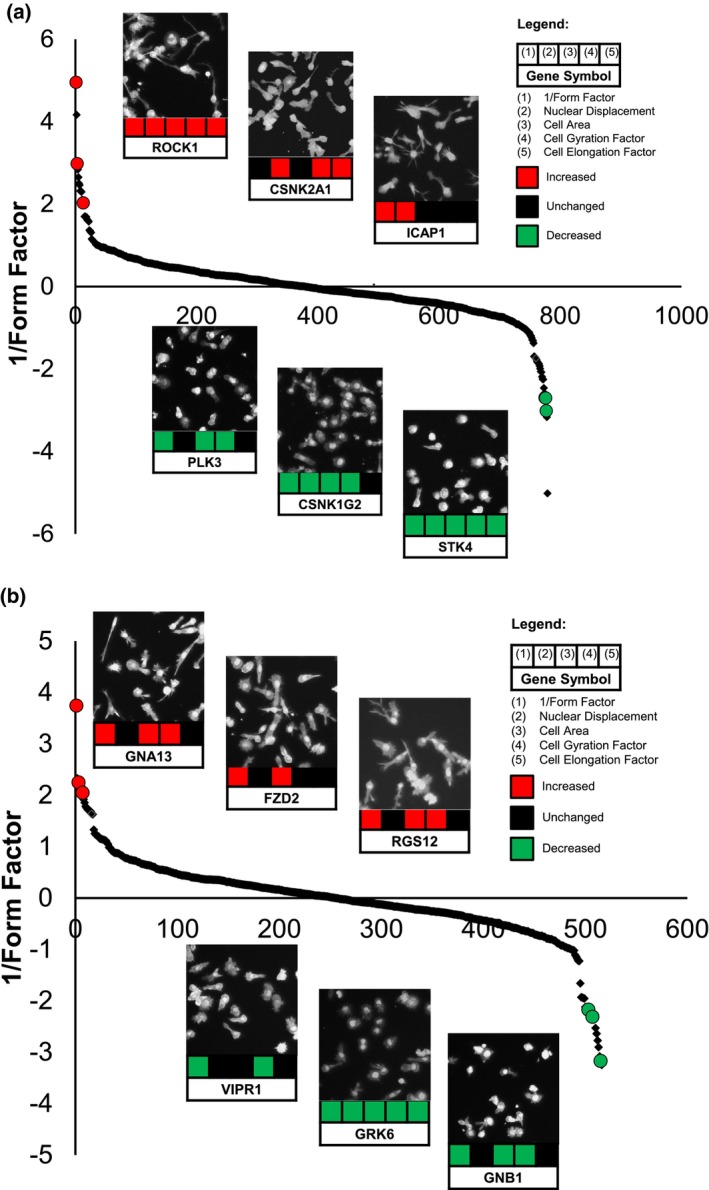
Representative hits from the RNAi library screens in HuT 78 T cells. **(a)** Representative hits from kinome RNAi library screening that increase (red), decrease (green) or have no effect (black) on LFA‐1‐mediated T cell polarization based on the 1/Form factor morphology parameter. The effect of these siRNAs on nuclear displacement, area, gyration radius and elongation factor morphology parameters are depicted below each image according to the legend in the Figure. **(b)** Representative hits from the GPCR/GPCR‐associated RNAi library screening that increase (red), decrease (green) or have no effect (black) on LFA‐1‐mediated T cell polarization based on the 1/Form factor morphology parameter is shown. Microscopy images in **a** and **b** were obtained by high content microscopy under 10× magnification.

After performing three replicate RNAi library screens targeting kinases and GPCR/GPCR‐associated proteins, siRNAs that significantly affected LFA‐1‐induced T‐cell polarization were classified as hits if one or more of the readouts for morphology (described above) were perturbed (Supplementary figure 1). siRNAs were only designated as hits if they exceeded the *Z*‐score threshold in at least 2 out of 3 screens. siRNAs from either library that resulted in similar morphological phenotypes were grouped together. From the RNAi library targeting kinases (719 genes), 58 siRNAs (8%) decreased T‐cell polarization significantly, typically resulting in cells with a rounder or less elongated morphology. For example, siRNAs targeting serine/threonine kinase (STK) 4, integrin‐linked kinase (ILK; a pseudokinase), Casein Kinase 1 isoform‐gamma 2, Polo‐like kinase 3 (PLK3) and p90 ribosomal S6 kinase alpha‐4 decreased LFA‐1‐mediated T‐cell polarization (Figure 2a and Supplementary figure 1). siRNAs targeting STK4 and ILK significantly reduced all five morphology parameters, whereas siRNAs targeting Casein Kinase 1 isoform‐gamma 2 reduced all parameters except for elongation factor. In contrast, siRNAs targeting PLK3 and p90 ribosomal S6 kinase alpha‐4 reduced three of the five morphology parameters (1/Form factor, gyration radius and area). STK4 is also known as Mst1 and has previously been implicated in chemokine‐mediated T‐cell polarization, adhesion to ICAM‐1 and motility,^23^, ^24^ whereas ILK, Casein Kinase 1 isoform‐gamma 2, PLK3 and p90 ribosomal S6 kinase alpha‐4 have not been reported in LFA‐1‐dependent polarization in T cells.

In contrast to these siRNAs which decreased the morphological phenotype of T cells, 70 siRNAs (9.7%) from the RNAi library targeting kinases resulted in an elongated or more spread‐like appearance. This included siRNAs targeting ROCK 1 and 2, myosin light chain kinase 3 (MLCK3), STK10 (also known as LOK), STK11 (also known as LKB1), STK17A (also known as DRAK1), integrin beta 1‐binding protein 1/ICAP‐1 (ITGB1BP1) and the catalytic and regulatory subunits of Casein Kinase 2 (CSNK2A1 and CSNK2B, respectively) (Figure 2a and Supplementary figure 1). The identification of ROCK1/2 and myosin light chain kinases from the screens is consistent with reports that siRNAs or pharmacological inhibitors targeting these kinase families result in an elongated phenotype when T cells are incubated on ICAM‐1‐coated surfaces or endothelial cells.^25^, ^26^ Interestingly, while we found that siRNAs targeting STK10 (LOK) increased T‐cell polarization on anti‐LFA‐1, T cells from STK10‐knockout mice were reported to polarize more efficiently and to migrate to a greater extent towards the chemokine SDF‐1α *in vitro* when compared with T cells from wild‐type mice.^27^ In contrast, STK11 (LKB1), STK17A (DRAK1), ICAP‐1 and CSNK2A1/CSNK2B have not been previously implicated in LFA‐1‐mediated T‐cell polarization and thus were novel hits from this RNAi library screen.

From the RNAi library targeting GPCR/GPCR‐associated proteins (390 genes), 39 siRNAs (10%) decreased T‐cell polarization significantly where at least one of the five morphology parameters was reduced (Figure 2b and Supplementary figure 1). For example, siRNAs targeting G protein‐coupled receptor kinase 6 (GRK6) significantly reduced all five morphology parameters resulting in a less elongated phenotype, whereas heterotrimeric G protein subunits Gβ1 and Gγ3 reduced three (1/Form factor, gyration radius and area) and two (1/Form factor and gyration radius) morphology parameters, respectively. In contrast, 45 siRNAs (11.5%) significantly increased at least one morphology parameter, typically resulting in an elongated phenotype. This included Frizzled‐2 (FZD2), the heterotrimeric G protein Gα_13_ (GNA13) and Regulator of G protein Signaling 12 (RGS12). (Figure 2b and Supplementary figure 1). Identification of GRK6 as a positive regulator of LFA‐1‐mediated T‐cell polarity correlates with a report that T cells from GRK6‐deficient mice are defective in transendothelial migration and/or chemotaxis towards SDF‐1α.^28^ Furthermore, our finding that siRNAs targeting Gα_13_ promote an elongated phenotype on anti‐LFA‐1 is consistent with T cells from Gα_12/13_ double‐knockout mice which display elongated uropods and an increase in cell polarity when plated on ICAM‐1.^29^ All other hits from this GPCR/GPCR‐associated protein RNAi library were novel and not previously demonstrated in T‐cell polarity and migration. In summary, 211 hits were revealed in these primary RNAi library screens using HuT 78 T cells plated on anti‐LFA‐1 and identified both known and novel components in LFA‐1‐mediated T‐cell polarity.

**Figure 3:**
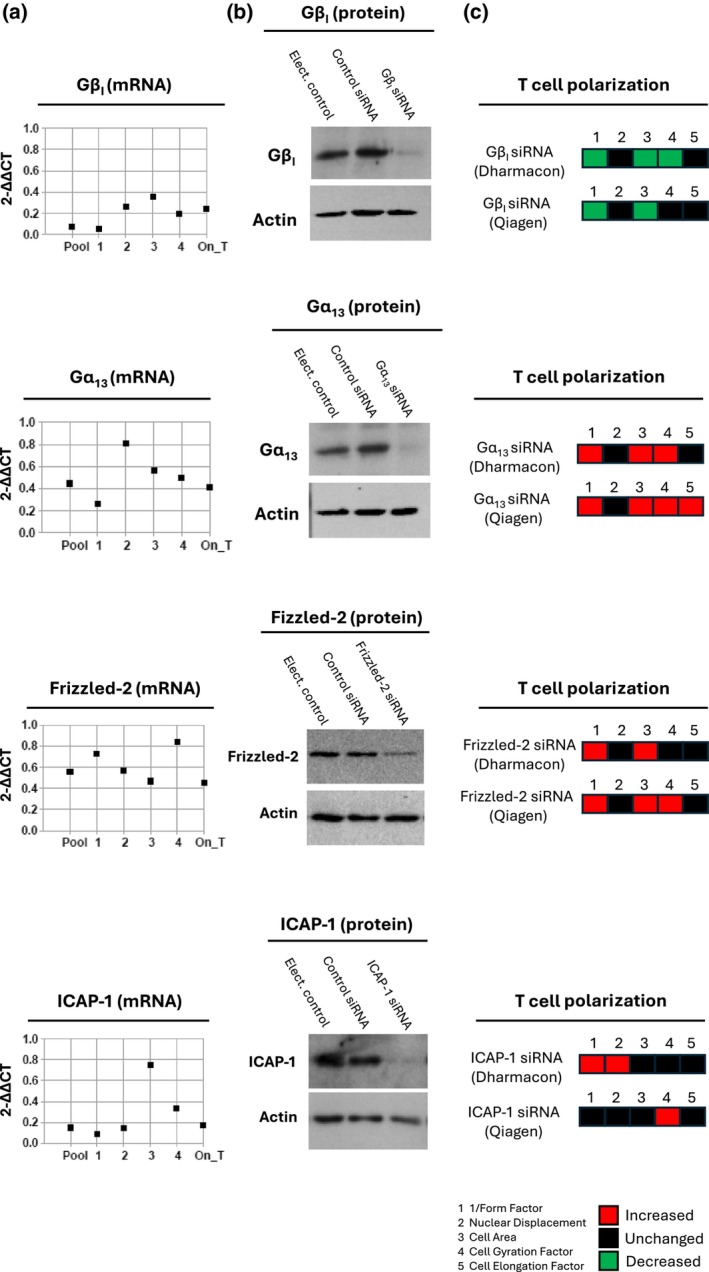
Validation of gene silencing of several hits from the RNAi library screens in the HuT 78 T cell line. **(a)** HuT 78 T cells were electroporated with 500 nM SMARTpool siRNAs (Pool), individual siRNAs 1–4 from the SMARTpools, or On‐Target Plus siRNAs (On_T; a pool of 4 siRNAs) targeting Gβ1, Gα13, Frizzled‐2 or ICAP‐1. The cells were electroporated with the appropriate non‐targeting control siRNA(s) in parallel. RNA was harvested from the cells after 72 h and RT‐qPCR was used to analyze gene expression. Target gene expression was normalized to GAPDH levels and represented as 2^−ΔΔCt^ whereby the control siRNA sample was set to a value of 1. **(b)** Western blotting for Gβ1, Gα13, Frizzled‐2 and ICAP‐1 expression in Hut 78 T cells 72‐h post electroporation with On‐Target Plus siRNAs (500 nM). **(c)** HuT 78 cells were electroporated with Qiagen siRNAs targeting Gβ1, Gα13, Frizzled‐2 or ICAP‐1. The cells were recovered after 72 h and incubated on anti‐LFA‐1 antibodies, followed by high content image analysis for T‐cell polarization. The T‐cell morphology data from the primary RNAi library screens (using Dharmacon SMARTpool siRNAs) was included as a comparison to the Qiagen siRNAs.

We selected several of the hits from the RNAi library screens for validation of gene silencing at the mRNA and protein level, namely Gβ1, Gα13, Frizzled‐2 and ICAP‐1. Silencing of gene expression at the mRNA level used the same SMARTpools that were used in the RNAi library screens as well as individual siRNAs 1–4 (Figure 3a). At least three of the four individual siRNAs demonstrated equivalent gene silencing as the SMARTpool siRNAs (Figure 3a). In addition, we confirmed gene silencing of the same targets at the mRNA level using the vendor's On‐Target Plus siRNAs (a pool of four siRNAs) which have reduced off‐target effects. The On‐Target Plus siRNAs were also used to verify knockdown of protein expression of Gβ1, Gα13, Frizzled‐2 and ICAP‐1 (Figure 3b). Finally, a set of independent pooled siRNAs targeting the same mRNAs were purchased from a different vendor and demonstrated similar effects on T‐cell polarization on anti‐LFA‐1 antibodies by high content image analysis (Figure 3c).

### Pathway analysis and molecular interaction networks reveals Gβ1 and Casein Kinase 2 as a novel regulator of T‐cell polarity and migration

**Figure 4:**
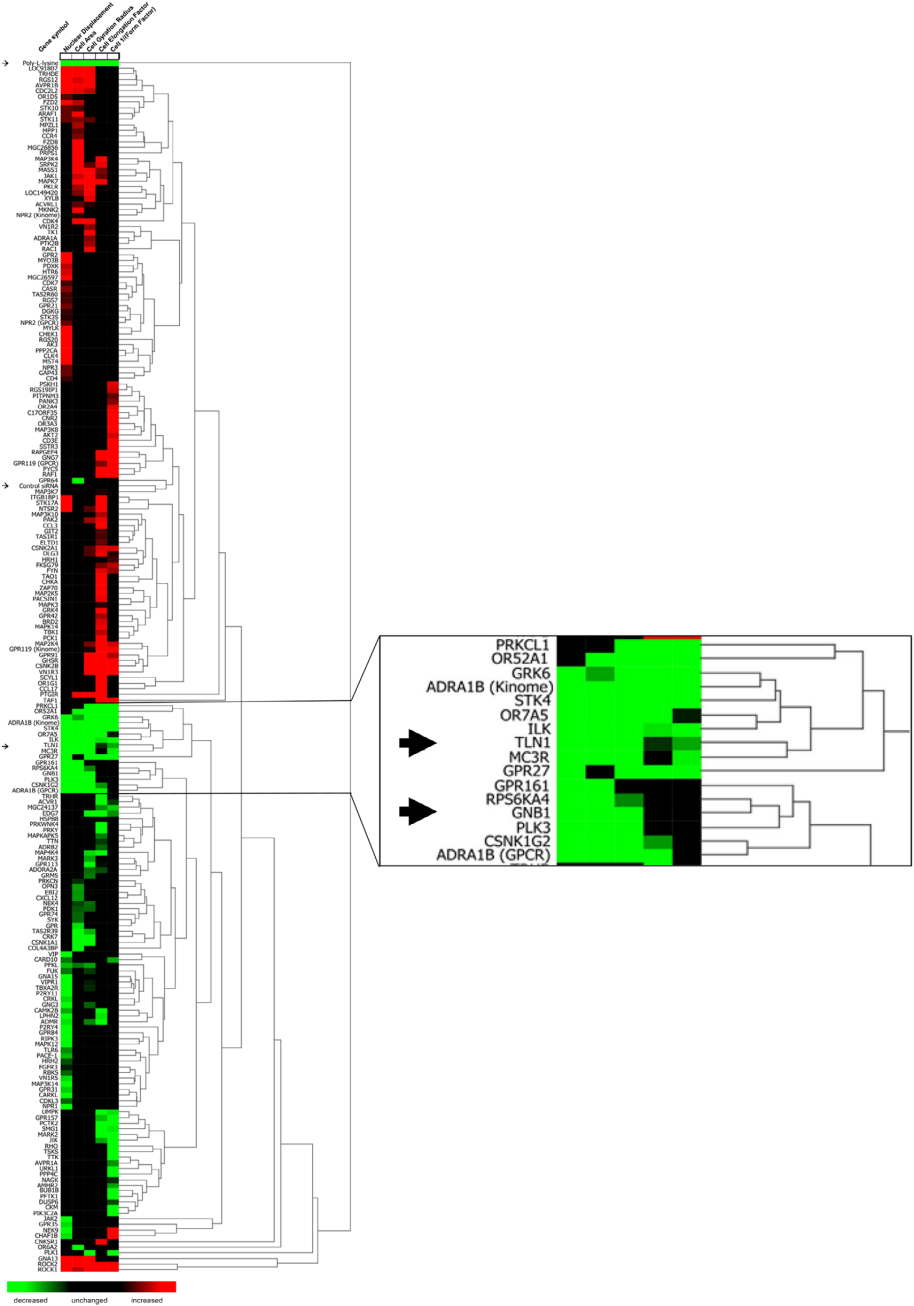
Hierarchical clustering of kinome and GPCR/GPCR‐associated RNAi library hits reveals common effects on cell morphology parameters. The magnified inset view of selected hits (arrows) indicates that silencing of talin‐1 and Gβ1 expression affects common morphology parameters, perhaps suggesting common roles in LFA‐1‐mediated T‐cell adhesion, polarization and migration.

Pathway analysis of the 211 hits identified from the siRNA screens revealed that the majority were involved in MAPK signaling (24 genes), neuroactive ligand–receptor interactions (23 genes), chemokine signaling (19 genes), calcium signaling (18 genes), the PI3‐kinase/Akt pathway (16 genes) and T‐cell receptor signaling (13 genes) (Table 1). Hierarchical clustering was then used to determine the inter‐relatedness of all hits identified in the screens (Figure 4). Interestingly, some hits from both the kinase and GPCR/GPCR‐associated protein RNAi libraries clustered with the talin‐1 phenotype (decreased polarity), whereas other hits clustered with the ROCK1/2 phenotype (increased polarity). For example, siRNAs targeting the kinases STK4, GRK6, ILK and PLK3 resulted in a phenotype that resembled T cells transfected with siRNAs targeting talin‐1. Similarly, siRNAs targeting the GPCR/GPCR‐associated proteins GPR27 and GβI also resulted in a phenotype that resembled T cells transfected with talin‐1. Clustering of similar morphological phenotypes may potentially provide insight into common/overlapping biological pathways and suggests that such genes may regulate LFA‐1‐mediated T cell polarity in a manner similar to talin‐1.

**Table 1:** Pathway analysis of the hits identified from the kinome and GPCR/GPCR‐associated RNAi library screens.

KEGG pathway	No. of genes	*P*‐value (FDR)	Associated genes
MAPK signaling pathway	24	2.18E−14	AKT2, CRKL, DUSP6, FGFR3, MAP2K4, MAP2K5, MAP3K4, MAP3K7, MAP3K8, MAP4K4, MAPK12, MAPK14, MAPK3, MAPK7, MAPKAPK5, MKNK2, PAK2, PRKX, RAC1, RAF1, RPS6KA4, STK4, TAOK1, TAOK3
Neuroactive ligand–receptor interaction	23	3.82E−13	ADORA2A, ADRA1A, ADRA1B, ADRB2, AVPR1A, AVPR1B, CNR2, GHSR, GPR35, GRM5, HRH1, HRH2, HTR6, LPAR3, MC3R, NPFFR2, NTSR2, P2RY4, PTGIR, SSTR3, TBXA2R, TRHR, VIPR1
Chemokine signaling pathway	19	1.39E−12	AKT2, CCL17, CCL3, CCR10, CCR4, CRKL, CXCL12, GNG3, GNG7, GRK4, GRK6, JAK2, MAPK3, PRKX, PTK2B, RAC1, RAF1, ROCK1, ROCK2
Calcium signaling pathway	18	9.60E−12	ADORA2A, ADRA1A, ADRA1B, ADRB2, AVPR1A, AVPR1B, CAMK2B, GNA15, GRM5, HRH1, HRH2, HTR6, MYLK, MYLK3, PRKX, PTK2B, TBXA2R, TRHR
Vascular smooth muscle contraction	17	2.81E−13	ADORA2A, ADRA1A, ADRA1B, ARAF, AVPR1A, AVPR1B, GNA13, MAPK3, MYLK, MYLK3, NPR1, NPR2, PRKX, PTGIR, RAF1, ROCK1, ROCK2
PI3K‐Akt signaling pathway	16	5.13E−06	AKT2, CDK4, FGFR3, GNG3, GNG7, JAK1, JAK2, LPAR3, MAPK3, PCK1, PKN1, PPP2CA, RAC1, RAF1, STK11, SYK
Platelet activation	14	1.39E−09	AKT2, FYN, GNA13, MAPK12, MAPK14, MAPK3, MYLK, MYLK3, PRKX, PTGIR, ROCK1, ROCK2, SYK, TBXA2R
T‐cell receptor signaling pathway	13	8.54E−10	AKT2, CD3E, CD4, CDK4, FYN, MAP3K7, MAP3K8, MAPK12, MAPK14, MAPK3, PAK2, RAF1, ZAP70
cGMP‐PKG signaling pathway	13	2.15E−07	ADRA1A, ADRA1B, ADRB2, AKT2, GNA13, MAPK3, MYLK, MYLK3, NPR1, NPR2, RAF1, ROCK1, ROCK2
Proteoglycans in cancer	13	5.19E−06	AKT2, ARAF, CAMK2B, FZD2, FZD8, MAPK12, MAPK14, MAPK3, PRKX, RAC1, RAF1, ROCK1, ROCK2
Pathways in cancer	13	0.000158	AKT2, ARAF, CDK4, CRKL, FGFR3, FZD2, FZD8, JAK1, MAPK3, RAC1, RAF1, STK4, TRAF1
Oxytocin signaling pathway	12	9.08E−07	CAMK2B, MAP2K5, MAPK3, MAPK7, MYLK, MYLK3, NPR1, NPR2, PRKX, RAF1, ROCK1, ROCK2
Focal adhesion	12	1.47E−05	AKT2, CRKL, FYN, ILK, MAPK3, MYLK, MYLK3, PAK2, RAC1, RAF1, ROCK1, ROCK2
Rap1 signaling pathway	12	1.55E−05	ADORA2A, AKT2, CRKL, FGFR3, LPAR3, MAPK12, MAPK14, MAPK3, PRKD3, RAC1, RAF1, RAPGEF4
Regulation of actin cytoskeleton	12	1.58E−05	ARAF, CRKL, FGFR3, GNA13, MAPK3, MYLK, MYLK3, PAK2, RAC1, RAF1, ROCK1, ROCK2
Ras signaling pathway	12	2.33E−05	AKT2, FGFR3, GNG3, GNG7, MAPK3, PAK2, PRKX, RAC1, RAF1, STK4, TBK1, ZAP70
Toll‐like receptor signaling pathway	11	2.03E−07	AKT2, CCL3, MAP2K4, MAP3K7, MAP3K8, MAPK12, MAPK14, MAPK3, RAC1, TBK1, TLR6
FoxO signaling pathway	11	8.37E−07	AKT2, ARAF, MAPK12, MAPK14, MAPK3, PCK1, PLK1, PLK3, RAF1, STK11, STK4
Adrenergic signaling in cardiomyocytes	11	3.53E−06	ADRA1A, ADRA1B, ADRB2, AKT2, CAMK2B, MAPK12, MAPK14, MAPK3, PPP2CA, PRKX, RAPGEF4
Tuberculosis	11	1.57E−05	AKT2, CAMK2B, JAK1, JAK2, MAPK12, MAPK14, MAPK3, PLK3, RAF1, SYK, TLR6
Epstein–Barr virus infection	11	3.24E−05	AKT2, CSNK2A1, JAK1, MAP2K4, MAP3K7, MAPK12, MAPK14, PRKX, SYK, TBK1, TRAF1
GnRH signaling pathway	10	4.81E−07	CAMK2B, MAP2K4, MAP3K4, MAPK12, MAPK14, MAPK3, MAPK7, PRKX, PTK2B, RAF1
Chagas disease (American trypanosomiasis)	10	9.08E−07	AKT2, CCL3, CD3E, GNA15, MAP2K4, MAPK12, MAPK14, MAPK3, PPP2CA, TLR6
Neurotrophin signaling pathway	10	4.40E−06	AKT2, CAMK2B, CRKL, MAP2K5, MAPK12, MAPK14, MAPK3, MAPK7, RAC1, RAF1
HTLV‐I infection	10	0.00107	AKT2, BUB1B, CD3E, CDK4, CHEK1, FZD2, FZD8, JAK1, MAP2K4, PRKX

**Figure 5:**
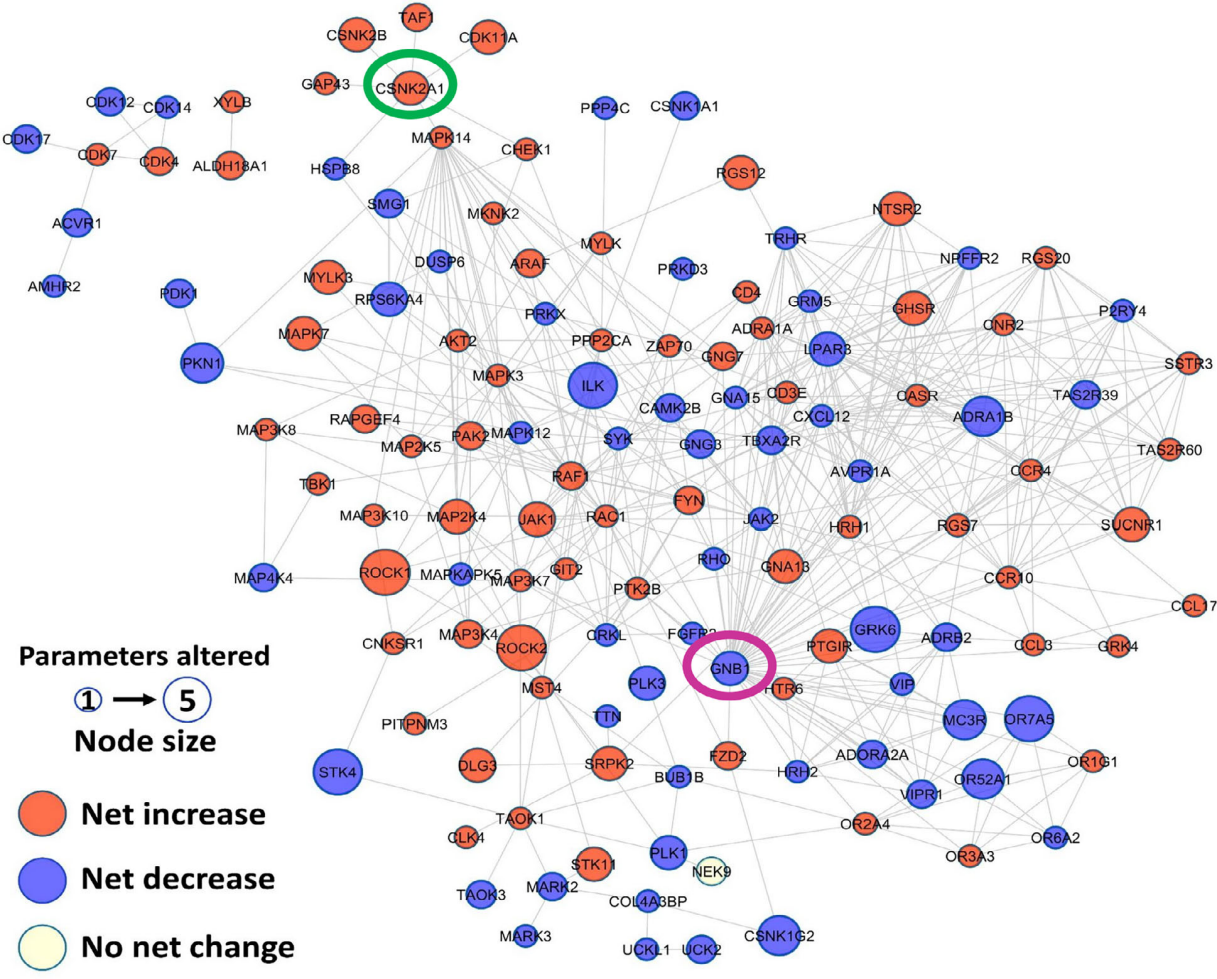
Interaction network of kinome and GPCR/GPCR‐associated RNAi library hits. The size of each node reflects the number of morphology parameters that were affected by this siRNA. Blue nodes indicate overall decreased morphology whereas red nodes indicate increased morphology. Gβ1 and Casein Kinase 2α are highlighted in purple and green, respectively.

**Figure 6:**
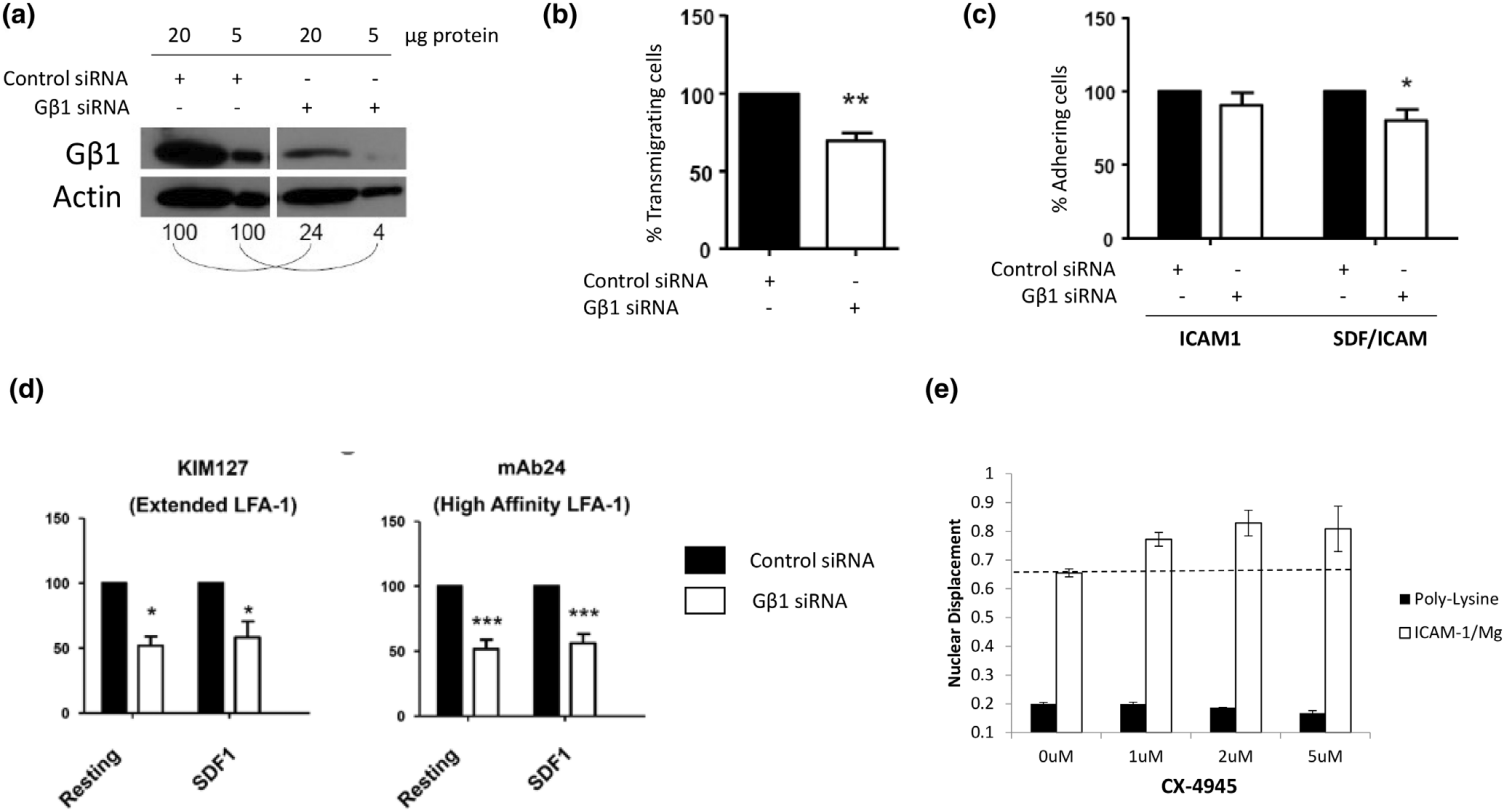
Validation of Gβ1 and Casein Kinase 2 as novel regulators of LFA‐1‐mediated T‐cell adhesion, polarity and migration in primary human T cells. **(a)** Confirmation of siRNA knockdown of Gβ1 expression in primary human T cells by Western blotting, with Actin used as a loading control. 20 μg or 5 μg of protein from control siRNA or Gβ1 siRNA treated primary human T cells was loaded per lane to ensure linearity of the Western blots. The numbers underneath the blots indicate the densitometry of the bands i.e. the band intensity for Gβ1 relative to the actin loading control was set at 100 for the control siRNA treated cells using either 20 μg or 5 μg of protein per lane. The band intensity for Gβ1 relative to the actin loading control in Gβ1 siRNA treated cells was calculated as a proportion of 100. **(b)** Knockdown of Gβ1 expression in primary human T cells perturbs migration in *in vitro* transwell assays in response to ICAM‐1 and SDF‐1α stimulation. **(c)** Knockdown of Gβ1 expression in primary human T cells affects adhesion to ICAM‐1 in the presence of SDF‐1α. **(d)** Knockdown of Gβ1 expression in primary human T cells inhibits the extended conformations of LFA‐1, indicated as a loss of binding of fluorescently labeled KIM127 (extended LFA‐1) or mAb24 (high affinity LFA‐1) reporter antibodies to T cells. The cells were analyzed by flow cytometry and gated on forward/side scatter. The mean fluorescence intensity (MFI) parameter was used to calculate the fluorescence intensity of reporter antibody binding and was expressed as a percentage of the control siRNA sample which was set at 100% **(e)** Treatment of primary human T cells with the Casein Kinase 2 inhibitor CX‐4945 (1–5 μM) increases T cell polarization on ICAM‐1. Statistical significance is indicated as * ≤ 0.05, ** ≤ 0.01 and *** ≤ 0.001.

We performed molecular network analysis of the combined hits from the library screens using Cytoscape to identify genes/proteins known to interact with one another based on experimentally validated studies (Figure 5). Such an approach enables the identification of genes/proteins that serve as central mediators within a known pathway (and may be assumed to play a more central role in T‐cell polarity), while highlighting others that are peripheral to a pathway and may play less important roles. Each hit from the siRNA screen was depicted as a node (circle) where the size of the node reflected the number of morphology parameters that were affected by this siRNA and color coded to reflect a decrease (blue) or an increase (red) in the overall parameters. Such analysis revealed that STK4 (a known positive regulator of lymphocyte polarity and adhesion^23^, ^24^) is peripheral to the network, interacting with Connector Enhancer of Kinase Suppressor of Ras 1 (CNKSR1) and TAO kinase 1 (TAOK1) identified in our screens. In contrast, the Gβ1 subunit is central to a network and demonstrates multiple interactions but has not been studied in the context of lymphocyte migration. It has, however, been shown to be required for neutrophil adhesion to ICAM‐1 and migration.^30^ Results from the primary RNAi screens in the HuT 78 T cell line demonstrated that siRNAs targeting Gβ1 expression resulted in a striking loss of cell polarity when stimulated with anti‐LFA‐1 antibodies. We subsequently confirmed silencing of Gβ1 protein by Western blotting in physiologically relevant primary human T cells (Figure 6a) and demonstrated that Gβ1 was required for migration through ICAM‐1‐coated filters towards SDF‐1 using transmigration assays (Figure 6b). Further experiments revealed that this defect in migration is likely due to an inability to adhere to ICAM‐1, as adhesion was significantly reduced following knockdown of Gβ1 (Figure 6c). Because affinity/adhesion to ICAM‐1 is mediated via at least two extended conformations on LFA‐1 that are induced via chemokine‐mediated inside‐out signaling (see Introduction), we investigated whether depletion of Gβ1 influenced these epitopes on LFA‐1 using fluorescently labeled monoclonal antibodies that detect the extended conformation of LFA‐1 (KIM127 antibody) or the high affinity confirmation of LFA‐1 (mAb24 antibody) by flow cytometry. We found that Gβ1 depletion reduced the basal and SDF‐1α‐mediated appearance of extended and high‐affinity confirmations on LFA‐1 (Figure 6d). Together, these studies highlight a novel role for Gβ1 in inside‐out signaling of LFA‐1 in T cells, with a corresponding defect in T cell adhesion, polarity and migration. This phenotype is similar to HuT 78 T cells depleted of talin, a phenotype that was suggested from our hierarchical clustering approach.

Another interesting cluster in this molecular network is the catalytic subunit of Casein Kinase 2 (CSNK2A1) interacting with its regulatory subunit (CSNK2B) and several other proteins including TATA‐Box Binding Protein Associated Factor 1 (TAF1), cyclin‐dependent kinase 11A (CDK11A), Growth Associated Protein 43 (GAP43), Mitogen‐Activated Protein Kinase 14 (MAPK14) and Checkpoint Kinase (CHEK1). Knockdown of each of these genes in the screening assays resulted in an increase in T cell polarity and thus revealed a similar morphological phenotype. Casein Kinase 2 is a ubiquitous serine/threonine kinase that functions as a tetramer in cells. The tetramer is composed of two catalytic domains, Casein Kinase 2α and/or Casein Kinase 2α' (encoded by CSNK2A1 and CSNK2A2 genes respectively), that tethers to two regulatory domains (CSNK2B).^31^ Interestingly, pharmacological inhibition of Casein Kinase 2 activity or genetic approaches to delete either the catalytic (α subunit) or regulatory domains inhibits the differentiation of naïve T cells into Th17 effector cells *in vitro*, with an increase in immunosuppressive regulatory T cells under Th17‐inducing conditions.^32^, ^33^, ^34^, ^35^, ^36^ Furthermore, pharmacological inhibition or genetic deletion of Casein Kinase 2 in T cells is protective in murine models of multiple sclerosis and inflammatory bowel disease, with a reduction in the infiltration of inflammatory TH17 T cells into the CNS or gastrointestinal tract and an increase in regulatory T cells.^32^, ^33^, ^34^, ^35^, ^36^ These latter studies suggest that, in addition to a role in regulation of T‐cell differentiation, Casein Kinase 2 may positively influence T‐cell migration and trafficking into tissues. Indeed, we confirmed that inhibition of Casein Kinase 2 activity in primary human T cells with CX‐4945, a well‐characterized pharmacological inhibitor of Casein Kinase 2 activity, increased T‐cell polarity on ICAM‐1 (the natural ligand of LFA‐1) in a manner similar to gene silencing in HuT 78 T cells (Figure 6e). These results uncover a novel role for this kinase in LFA‐1‐mediated T‐cell polarization. Overall, these studies in primary human T cells validate both Gβ1 and Casein Kinase 2 as novel regulators of T‐cell polarity and migration.

### The β_I_ integrin‐associated protein ICAP‐1 is a novel regulator of β2/LFA‐1‐mediated T‐cell migration

**Figure 7:**
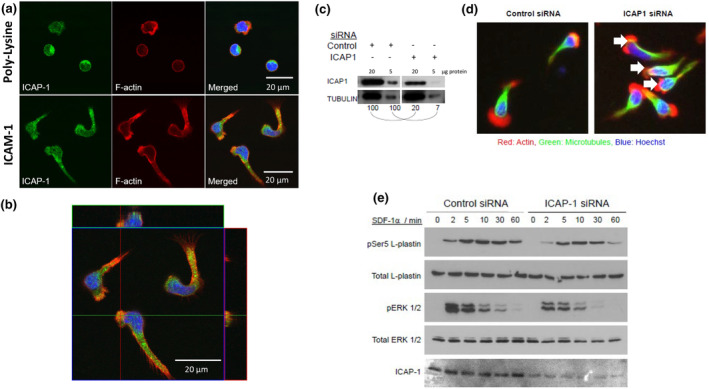
ICAP‐1 redistributes to the leading edge and trailing tail of migrating primary human T cells and regulates T‐cell polarity in response to ICAM‐1 stimulation. **(a)** Intracellular localization of ICAP‐1 in non‐polarized and polarized human T cells. ICAP‐1 partially co‐localizes with F‐Actin in migrating T cells. Images were obtained by confocal microscopy. **(b)** Z‐stack confocal image of ICAP‐1 and F‐Actin co‐localization in migrating human T cells. **(c)** Confirmation of siRNA knockdown of ICAP‐1 expression in primary human T cells by Western blotting, with actin used as a loading control. 20 μg or 5 μg of protein from control siRNA or ICAP‐1 siRNA treated primary human T cells was loaded per lane to ensure linearity of the western blots. The numbers underneath the blots indicate the densitometry of the bands i.e. the band intensity for ICAP‐1 relative to the actin loading control was set at 100 for the control siRNA treated cells using either 20 μg or 5 μg of protein per lane. The band intensity for ICAP‐1 relative to the actin loading control in ICAP‐1 siRNA treated cells was calculated as a proportion of 100 **(d)** Effect of ICAP‐1 knockdown on ability of T cells to produce leading edges in response to ICAM‐1 stimulation. Microscopy images were obtained by high content microscopy under 10× magnification. **(e)** ICAP‐1 expression influences the phosphorylation of the actin‐bundling protein L‐plastin.

**Figure 8:**
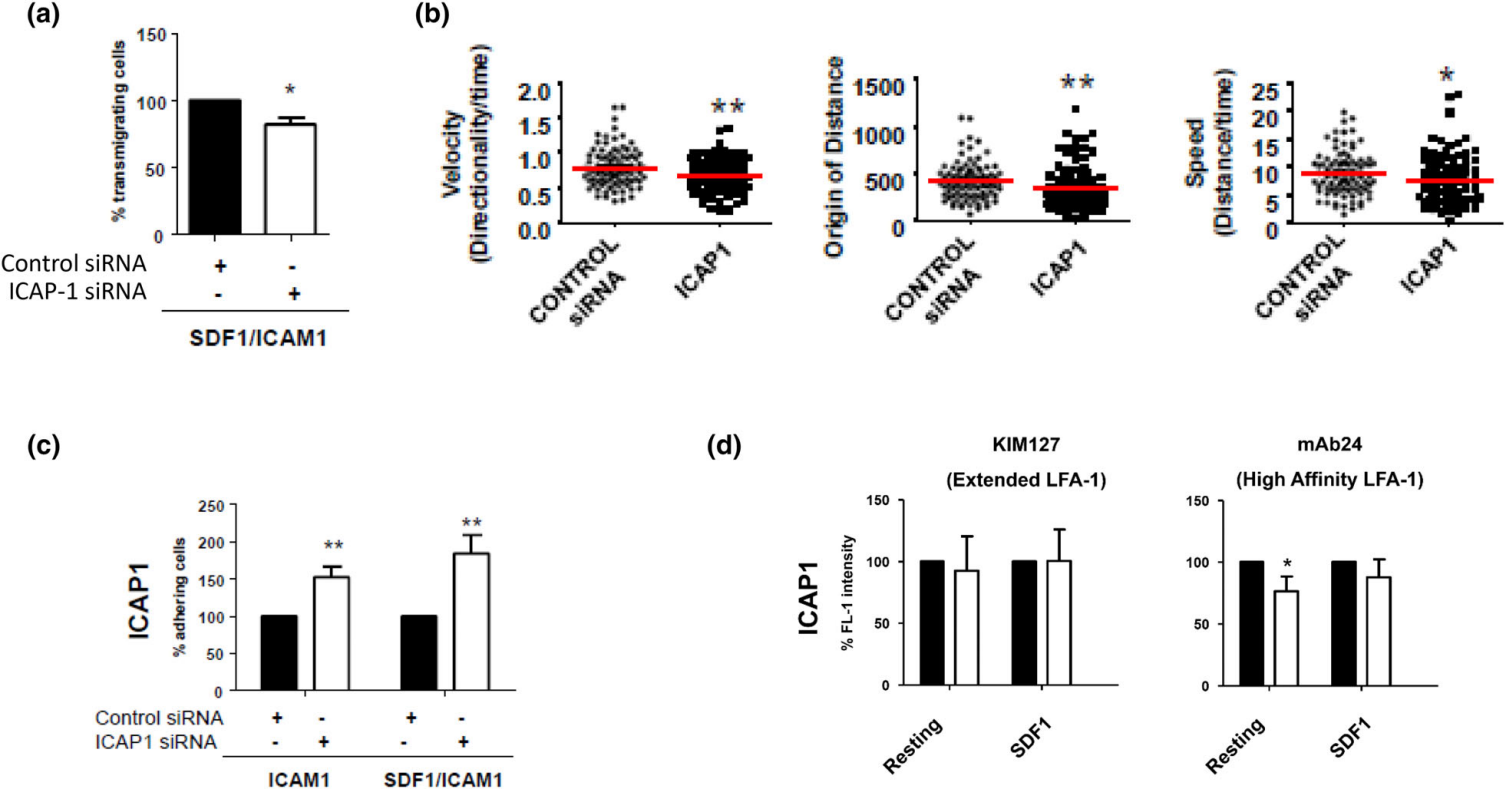
Validation of ICAP‐1 as a novel regulator of LFA‐1‐mediated T‐cell adhesion, polarity and migration in primary human T cells. **(a)** Knockdown of ICAP‐1 expression in primary human T cells perturbs migration in *in vitro* transwell assays in response to ICAM‐1 and SDF‐1α stimulation. **(b)** Knockdown of ICAP‐1 expression in primary human T cells perturbs T‐cell migration. **(c)** Knockdown of ICAP‐1 expression in primary human T cells increases adhesion to ICAM‐1 in the presence of SDF‐1α. **(d)** Knockdown of ICAP‐1 expression in primary human T cells does not affect the extended conformations of LFA‐1. Statistical significance is indicated as * ≤ 0.05 and ** ≤ 0.01.

The pathway analysis performed above defines regulatory networks between experimentally validated genes/proteins but may overlook genes/proteins that do not interact with any other partners identified in our screens. One such notable example was ICAP‐1 (ITGB1BP1), which is an adaptor protein that negatively regulates β1 integrins,^37^ and which did not form networks with any other genes/proteins identified in the RNAi screens. We found that siRNAs targeting ICAP‐1 perturbed cell polarity in the HuT 78 T cells screens, resulting in a loss of a defined leading edge and the appearance of a disorganized trailing tail/uropod. This loss in cell polarity was reflected as an increase in several morphological parameters predominantly 1/Form factor and nuclear displacement. We investigated the subcellular distribution of ICAP‐1 in resting and migrating primary human T cells by confocal microscopy. ICAP‐1 did not co‐localize with F‐actin in resting cells (Figure 7a). However, primary human T cells stimulated to migrate on ICAM‐1 showed a striking redistribution of F‐actin to the leading edge of the cell with partial co‐localization with ICAP‐1 that predominantly localized to a region just behind the leading edge and throughout the uropod (Figure 7a). Z‐stack analysis confirmed the lack of co‐localization of ICAP‐1 with F‐actin at the leading edge (Figure 7b). We then looked at the role of ICAP‐1 in LFA‐1‐mediated T‐cell polarity in more detail. Western blotting also demonstrated depletion of ICAP‐1 protein in primary human T cells transfected with siRNAs (Figure 7c). Primary human T cells that were depleted of ICAP‐1 did not generate defined leading edges when triggered to migrate on ICAM‐1 (Figure 7d). We have previously shown that the actin‐bundling protein L‐plastin regulates the ability of T cells to polarize and migrate in response to stimulation with the chemokine (SDF‐1α) stimulation.^38^ Furthermore, phosphorylation of Ser5 regulates actin bundling^39^ and is induced by SDF‐1α in primary human T cells.^38^ Using phosphorylation of Ser5 on L‐plastin as a readout of T cell polarity, we evaluated whether ICAP‐1 depletion affected this pathway. Depletion of ICAP‐1 in primary human T cells resulted in lower levels of phosphorylated L‐plastin at Ser5 compared with control cells following stimulation with SDF‐1α (Figure 7e). Phosphorylation of ERK 1/2 was also perturbed following ICAP‐1 depletion. These studies clearly demonstrate that ICAP‐1 expression is required for T‐cell polarity. We next determined whether this loss of polarity manifested as a defect in migration/motility. Depletion of ICAP‐1 expression in primary human T cells reduced migration in a transwell assay in response to ICAM‐1 and SDF‐1α stimulation (Figure 8a). In addition, live cell migration assays determined that ICAP‐1 depletion significantly reduced cell velocity, origin of distance and speed (Figure 8b). This defect in migration appeared to be due to increased adhesion to ICAM‐1 both in the absence or presence of SDF‐1α (Figure 8c). Hence, ICAP‐1 negatively regulates T cell adhesion to ICAM‐1. Unlike Gβ1 depletion which resulted in a loss of polarity, migration and LFA‐1 activation, depletion of ICAP‐1 did not affect the appearance of the extended or high‐affinity epitopes on LFA‐1 (Figure 8d). This suggests that ICAP‐1 influences T‐cell adhesion to ICAM‐1 independently of mechanisms that affect LFA‐1 affinity (see Discussion).

## DISCUSSION

In this study, we have identified over 200 putative GPCR/GPCR‐associated proteins and kinases that modulate LFA‐1‐mediated T‐cell polarity in the HuT 78 T‐cell line using a high content image‐based, fixed‐cell assay. T‐cell polarity was used as a surrogate for migration given that it is now well established that the formation of a polarized morphology, characterized by a leading edge and trailing tail, is necessary for efficient migration.^40^ We demonstrated previously that analysis of T‐cell polarity using five independent parameters focusing on cell size, shape, complexity of periphery and nucleus position was sufficient to detect a variety of T‐cell morphologies.^19^ Using this image‐based assay in conjunction with RNAi library screening identified several genes that have been previously reported to influence T‐cell polarity and/or migration, as well as numerous other novel genes that hitherto have not been implicated in T‐cell polarity/migration. Hence, the identification of genes that are known to influence T‐cell adhesion and migration validates our screening approach, while the identification of novel genes/proteins provides a comprehensive resource for further exploration in the context of T‐cell polarity and migration.

siRNAs that resulted in the most striking loss of cell polarization where all five morphology parameters were reduced included the serine/threonine kinase STK4 (Mst1) and GRK6, both of which have known roles in T cell adhesion and migration.^23^, ^24^, ^28^ Similarly, siRNAs targeting ILK and olfactory receptor family 7 (OR7A5) also resulted in similar phenotypes but this pseudokinase and GPCR‐associated kinase, respectively, have not been implicated in LFA‐1‐mediated migration and were novel hits. In contrast, siRNAs that promoted an elongated phenotype in HuT 78 T cells when stimulated through LFA‐1 where all five morphology parameters were increased included ROCK1/2, both of which have purported roles in T‐cell polarity and migration.^25^, ^26^ A further 205 siRNAs targeting various kinases and GPCR/GPCR‐associated proteins were identified in our screening approach and thus a major challenge when undertaking such assays is selecting the number of hits to follow up and validate in further assays. A combination of hierarchical clustering and/or network analysis highlighted that Gβ1 (GNB1) and Casein Kinase 2 (CSNK2A1/CSNK2B) may have central roles in T‐cell polarity, adhesion and migration and were chosen for further study on that basis, whereas ICAP‐1 (ITGBP1) was selected for further analysis based on its known role in β_1_ integrin migration^37^ and potentially novel role(s) in β_2_ integrin function from our RNAi library screening studies. A limitation of our RNAi screening approach is that a crosslinking antibody specific for the α_L_ chain of the LFA‐1 integrin was used to stimulate T‐cell polarization and migration. However, we have previously demonstrated that stimulation of the HuT 78 T cell line with this antibody promotes the acquisition of a motile phenotype and the appearance of well‐defined leading edges and uropods, similar to primary human T cells when stimulated with the natural ligand ICAM‐1.^6^ Importantly, we validated roles for Gβ1, Casein Kinase 2 and ICAP‐1 from the RNAi library screening assays in physiologically relevant primary human T cells in migration and adhesion assays using the natural ligand ICAM‐1. In addition, while the polarization assays on anti‐LFA‐1 were carried out after 72 h following siRNA delivery, it is conceivable that some proteins may have a slower turnover and thus these targets may not have been depleted in our study. We chose this 72‐h timepoint based on talin‐1 knockdown and confirmed that four other targets, namely Gβ1, Gα13, Frizzled‐2 and ICAP‐1, were depleted in HuT 78 T cells at both the mRNA and protein level thus validating our approach (Figure 3).

We identified the heterotrimeric subunit Gβ1 subunit as a positive regulator of LFA‐1 integrin affinity and adhesion to ICAM‐1 in primary human T cells, and identified a role for this subunit in ICAM‐1‐directed polarity and migration. Heterotrimeric G proteins are composed of α/β/γ subunits that transduce key intracellular signals downstream of chemokine receptors, and thus are involved in the regulation of integrin activation.^41^ To the best of our knowledge, this is the first report for a role for Gβ1 in T‐cell adhesion, polarity and migration. However, a similar role has been described in neutrophils, where silencing of Gβ1 reduced the chemokine‐induced high affinity conformation of LFA‐1 leading to a concomitant loss in migration *in vitro* and *in vivo*.^30^ In that study, Gβ1 was shown to activate the small GTPase Rac1 that regulates actin polymerization. In contrast, the heterotrimeric G proteins Gα_12/13_ negatively regulate LFA‐1 activation in human CD4^+^ T cells, as genetic deletion of these subunits results in increased polarity on ICAM‐1, a loss in migratory activity and increased Rac1 GTP loading.^29^ Notably, our study here also identified Gα_13_ as a negative regulator of T‐cell polarity when stimulated through the LFA‐1 integrin. Collectively, these studies in human immune cells highlight both positive and negative roles for heterotrimeric G proteins in T‐cell function and underscores their opposing roles in integrin activation, T cell adhesion and migration.

Casein Kinase 2 was identified as a novel regulator of T cell polarity in our RNAi screening strategy, as siRNAs targeting either the catalytic domain (CSNK2A1) or regulatory domain (CSNK2B) increased cell spreading when stimulated through the LFA‐1 integrin. This phonotype was confirmed in primary human T cells treated with a well‐characterized pharmacological inhibitor of Casein Kinase 2. It is interesting to note that a reduction in T cell infiltration into the central nervous system and gastrointestinal tract has been reported following pharmacological inhibition of Casein Kinase 2 or genetic deletion of the catalytic or regulatory domains genes in T cells.^32^, ^33^, ^34^, ^35^, ^36^ Our results presented in this study supports the view that Casein Kinase 2 is required for T‐cell polarity. Given that this kinase is also required for the differentiation of naïve CD4^+^ T cells into Th17 effector cells, Casein Kinase 2 may serve as a therapeutic target for the treatment of inflammatory diseases including multiple sclerosis and inflammatory bowel disease.

The third gene that we characterized in detail in primary human T cells was ICAP‐1. ICAP‐1 is a β1‐integrin interacting protein, binding via a unique NPXY motif located on the cytoplasmic domain. This is a unique interaction as ICAP‐1 binding was originally reported as being selective for β1‐integrins and not β2‐integrins including LFA‐1.^42^ ICAP‐1 competes with talin for β1‐integrin and negatively regulates activity/avidity, focal adhesion formation and cell spreading on fibronectin. The role of ICAP‐1 in T cell migration has not been investigated and therefore we were intrigued by the profound effect on LFA‐1‐mediated T‐cell polarity when ICAP‐1 expression was knocked down in primary human T cells. Further experiments demonstrated that T cells lacking ICAP‐1 expression have a disorganized actin cytoskeleton manifesting with an irregular actin cap at the leading edge. This was mirrored by live cell migration experiments where decreased velocity, origin of distance and speed were observed when the cells were plated on ICAM‐1. Mechanistically, we have demonstrated that the phenotype associated with loss of ICAP‐1 is caused by blunted phosphorylation of the actin bundling protein L‐plastin, which we described as a novel regulator of directed T‐cell polarity and migration.^38^ Interestingly, ICAP‐1 depletion in primary human T cells resulted in increased adhesion to ICAM‐1, a phenotype that mirrors increased adhesion to β1‐integrin ligands in non‐immune cells. This implies that ICAP‐1 is a negative regulator of LFA‐1 in human T cells. However, increased adhesion to ICAM‐1 following ICAP‐1 depletion was not due to an increase in the extended or high affinity conformations of LFA‐1. Interestingly, a similar phenotype was reported in T cells where the WNK1 kinase was identified as a negative regulator of β2 integrins but in a manner that was independent of LFA‐1 affinity status.^43^ Here the authors demonstrated a profound increase in Rap1 activity following WNK1 depletion which the authors proposed contributes to LFA‐1 function and adhesion/migration. This suggests that ICAP‐1 may also negatively regulate LFA‐1 in T cells via Rap1 signaling, although this remains to be tested. While it is intriguing that ICAP‐1 influences LFA‐1 function in human T cells, this was supported by biochemical assays which demonstrated that ICAP‐1 was associated with the β2 integrin chain, as well as the β1 integrin chain, in detergent extracted complexes isolated from primary human T cells. This suggests that ICAP‐1 may interact directly or indirectly with LFA‐1 (Supplementary figure 2).

Several studies have performed RNAi library screening or CRISPR screening to identify regulators of T‐cell migration *in vitro* and *in vivo*. Tybulewicz and colleagues knocked down the expression of 719 kinase and kinase‐related genes in the Jurkat leukemic T‐cell line to identify regulators of adhesion to antigen‐presenting cells and migration.^43^ WNK1 was identified as a negative regulator of integrin‐mediated adhesion and was required for T‐cell migration *in vitro* and *in vivo*. Kendirli *et al*.^44^ conducted a genome‐wide *in vivo* CRISPR screen to identify key genes that modulate T‐cell infiltration into the CNS using rat models. Eighteen genes, including the kinase GRK2 and the heterotrimeric G protein Gαi2, were identified as being essential for promoting T‐cell entry into the CNS. Similarly, Rogers *et al*.^45^ used the Sleeping Beauty mutagenesis system to identify candidate genes mediating T‐cell infiltration into tumors. Over 400 genes were identified in these studies conducted across three tumor models. Recently, Johansen *et al*.^46^ utilized CRISPR screening in murine T cells to target 570 known and potential PtdIns(3,4,5)P_3_‐binding proteins that influence T‐cell binding to ICAM‐1. While there is little overlap in terms of common genes between all of these screens (including the screen described here), this may be due to the different cell types used (HuT 78 vs Jurkat vs primary mouse T cells), different assay readouts (polarization vs ICAM‐1 binding vs infiltration into the CNS) and different strategies to perturb gene expression (siRNA vs Sleeping Beauty vs CRISPR). Although T‐cell lines such as HuT 78 and Jurkats have limitations when compared with primary human T cells,^47^ they are ideal for screening of RNAi libraries due to their uniformity, relative ease in transfection, ease of handling and efficient scale‐up. Both we and Kochl *et al*.^43^ conducted our screens in T‐cell lines and thereafter confirmed the phenotypes in physiologically relevant primary human T cells, which validates the use of such cell lines for RNAi library screening. In summary, the study described here highlights Gβ1, Casein Kinase 2 and ICAP‐1 regulating T‐cell polarity and migration and suggests new therapeutic targets for the treatment of inflammatory diseases. Furthermore, this study also highlights numerous other interesting molecules that should be studied in more detail in the context of T‐cell migration that will ultimately provide a more comprehensive understanding of the mechanisms regulating LFA‐1 function in T cells.

## METHODS

### Chemicals and reagents

The recombinant human ICAM‐1/Fc chimera was purchased from R&D Systems (Oxford, UK). Recombinant human SDF‐1α (CXCL12) and IL‐2 were from Peprotech (London, UK). The CellTiter‐Blue Viability Assay was from Promega (Madison, USA). Monoclonal anti‐α_L_ antibody (clone SPV‐L7) specific for LFA‐1 was purchased from Monosan (Uden, The Netherlands). Rabbit anti‐human ICAP‐1 antibody was a kind gift of Corinne Albigès‐Rizo (Institut Albert Bonniot, Grenoble, France). Hoechst 33258 and Hoechst 33342 were from Life Technologies (Paisley, Scotland). Goat anti‐mouse IgG, goat anti‐human IgG (Fc specific), phytohemaggluttinin (PHA), phalloidin‐TRITC, monoclonal anti‐talin‐1, monoclonal anti‐α‐tubulin and rabbit anti‐α‐actin were purchased from Sigma (St Louis, USA). Antibodies to Gβ1, Gα13 and β1 integrin was purchased from Santa Cruz Biotechnology (California, USA). Rabbit anti‐human Frizzled‐2 was from Zymed Laboratories (San Francisco, USA). Antibodies to phosphorylated and total ERK 1/2, HRP‐conjugated anti‐mouse IgG and anti‐rabbit IgG were purchased from Cell Signaling Technology (Danvers, USA). The antibody that recognizes L‐plastin when phosphorylated at Ser5 was generously provided by Dr Eric Brown (Genentech, San Francisco, USA). Anti‐pan L‐plastin antibody (clone 4A) was obtained from Thermo Fisher Scientific (Fremont, USA). AlexaFluor 488 goat anti‐mouse IgG was purchased from Invitrogen (Massachusetts, USA). The β2 integrin reporter antibodies KIM127 and mAB24 were kindly provided by Professor Nancy Hogg (Cancer Research UK London Research Institute, London, UK). CX‐4945 was purchased from MedChem Express (New Jersey, USA). All other chemicals were purchased from Sigma (St Louis, USA).

### RNAi libraries and siRNAs

The siGenome™ RNAi libraries targeting the Kinome (G‐003500) and GPCR/GPCR‐associated proteins (G‐003600) were obtained from Dharmacon (Colorado, USA) and were used for the primary screens. These libraries were provided in SMARTpool format and therefore contained a mixture of four distinct siRNAs targeting each mRNA. SMARTpool siRNAs, individual siRNAs 1–4 from the SMARTpools and On‐TARGETplus siRNAs targeting Gβ1, Gα13, Frizzled‐2 and ICAP‐1 were also obtained from Dharmacon and were used for validation studies. A set of independent single siRNAs targeting these same genes were obtained from Qiagen (Hilden, Germany).

### Cell culture

The cutaneous CD4^+^ T lymphoma cell line HuT 78 (catalogue number TIB 161) was obtained from the American Type Culture Collection (Manassas, USA) and cultured with RPMI‐1640/L‐Glutamine/Hepes supplemented with 10% fetal calf serum, 1.5% sodium bicarbonate and 1 mM sodium‐pyruvate (Complete growth medium; all from Gibco Life Technologies, Paisley, Scotland). The cells were maintained at 0.1–1 × 10^6^ cells mL^−1^ in a humidified atmosphere at 37°C and 5% CO_2_. New cell stocks from the same batch were re‐cultured for each replicate RNAi screen. Primary human T cells were isolated from buffy‐coat blood packs provided by the Irish Blood Transfusion Service (St James's Hospital, Dublin, Ireland) and expanded with PHA and IL‐2 as described previously.^48^ Primary human T cells were used for experiments between days 5 and 12 after culturing in IL‐2‐supplemented RPMI‐1640 containing 10% FCS and antibiotics.

### Electroporation and nucleofection

The 2–3‐day‐old HuT 78 T cell cultures were harvested and re‐suspended at a density of 1 × 10^6^ cells mL^−1^ in complete growth medium. Then 0.75 × 10^6^ cells were delivered automatically to source 96‐well plates containing 500 nM siRNA using a microdispenser. The siRNA/cell mixture was then transferred to 2 mm or 4 mm gap high‐throughput 96‐well electroporation plates (BTX, Harvard Apparatus, Holliston, USA). The cells were electroporated three times at 200 V (for 2 mm gap) or 350 V (for a 4‐mm gap) with a BTX ECM 830 square‐wave electroporator (Harvard Apparatus, Holliston, USA) using a pulse length of 2 ms. An interval time of 4 min was left between each pulse. Electroporated cells were recovered and gently resuspended at 0.1–0.2 × 10^6^ cells/well in complete growth medium and incubated in standard Nunc 96‐well plates. The cells were cultured in a sealed humidified incubator for 72 h at 37 °C to allow silencing of gene expression. The same electroporation conditions and apparatus were used for silencing of gene expression in HuT 78 cells using single 2‐mm gap cuvettes (Harvard Apparatus), albeit that the cells were recovered in 12‐well plates containing 1 mL of complete growth media. PHA/IL‐2‐expanded primary human T cells were transfected with On‐TARGETplus siRNAs (Dharmacon) using the Lonza (Amaxa) nucleofection system as described previously.^49^

### Anti‐LFA‐1 T cell migration assay

Migration assays from individual RNAi screens were set‐up in triplicate. Standard Nunc 96‐well plates (Thermo Fisher Scientific, Waltham, USA) were first coated with 5 μg mL^−1^ goat anti‐mouse IgG diluted in sterile PBS and incubated overnight at 4°C. The plates were washed with sterile PBS and incubated with an antibody specific for the α_L_ integrin chain (1/1000 dilution of clone SPV‐L7 in sterile PBS) for 2–3 h at 37°C. After washing the wells with sterile PBS, the cells were transferred to the antibody‐coated migration plates at a density of 6000–7500 cells/well using an electronic multichannel pipette. The cells were allowed to migrate on the antibody substrate for 4 h at 37°C. Migration was stopped with a paraformaldehyde fixation solution (final concentration 4%) for 45 min at room temperature. Following a blocking and permeabilization step (5% BSA, 0.1% Triton‐X100 in PBS), the cells were stained with Hoechst 33258 (1 μg mL^−1^) and phalloidin‐TRITC (3 μg mL^−1^) to label the nucleus and F‐actin, respectively. The wells were washed with PBS, stored at 4°C and protected from light until image acquisition and analysis.

### Analysis of anti‐LFA‐1 T cell migration

T cell migration was quantified using an image‐based high content analysis platform (IN Cell Analyzer 1000, GE Healthcare, Illinois, USA). Images were acquired with a 10× objective using a trichroic filter set. Eight fields were acquired per well, therefore giving a total of 24 fields for every siRNA SMARTpool per screen. Morphological analysis was performed using IN Cell Investigator 3.6 software (GE Healthcare), which identifies cells by nuclear dye staining. The morphology of the cells was quantified using the Morphology 1 package software and was based on phalloidin‐TRITC staining of the F‐actin cytoskeleton. The morphological parameters 1/Form factor, nuclear displacement, cell area, cell gyration radius and cell elongation were chosen as read‐outs of migration‐related morphological changes, as we have previously shown these to be informative in T‐cell polarity/migration assays.^19^ Raw data were normalized to Control siRNA per siRNA plate (6 samples). Identification of hits from the RNAi screens were calculated using *Z* scores.^50^ The *Z* score expresses a sample value as the distance from the mean sample value in multiples of the standard deviation and is defined to be

$$Z\;\text{score}=\left(x-\text{mean}\right)/\text{STDEV}$$

where *x* = is an individual value for a parameter, mean = the mean value of all samples for that parameter and STDEV is the standard deviation of all samples for that parameter. *Z* scores were calculated for individual plates from the screens. Effects mediated by specific siRNAs were defined as statistically significant if they displayed a *Z*‐score value of ±1.5 in at least two out of the three siRNA screens.

### Hierarchical clustering

Hierarchical clustering was performed as described^51^ with average linkage over the different parameters' scored values in the three different experiments. The scores of the controls (control siRNA, TLN1 and poly‐l‐lysine) were averaged per experiment. The computation was performed with KNIME 2.5.4, and HiTS (0.5.4 main and 0.4.1 experimental versions) for visualization. Leaf ordering was used for optimal visual result.^52^

### Protein–protein interaction (PPI) network analysis

Protein–protein interaction (PPI) networks were generated within Cytoscape (version 3.01).^53^ All interaction data were obtained from STRING ver 9.05 using the data import function through CluePedia ver 1.07. Only experimentally validated and curated interactions were used to build PPI networks. Gene Ontology (GO) function enrichment for downstream target genes was performed in Cytoscape using the ClueGo app (version 2.07) (Cordeliers Research Center, Paris, France).^54^

### Transwell assays

Transwell chemotaxis assays were carried out in triplicate using 96‐well ChemoTX migration plates (Neuroprobe, Gaithersberg, USA) with a 3‐μm pore size filter. The upper sides of the filters were coated with 5 μg mL^−1^ goat anti‐human IgG (Fc‐specific) and incubated at 4°C overnight. After washing with sterile PBS, the filters were coated with recombinant human ICAM‐1/Fc (2 μg mL^−1^) and incubated at 37°C for 2 h. Unbound ICAM‐1/Fc was then removed and the filters were washed with sterile PBS. Primary human T cells were deprived of IL‐2 and growth factors by re‐suspending the cells at 2.5 × 10^6^ cells mL^−1^ in RPMI‐1640 supplemented in 0.5% BSA for 2 h at 37°C. A volume of 25 μL of cells was then placed on the upper side of the filters. SDF‐1α (100 ng mL^−1^ in 0.5% BSA/RPMI‐1640) was used as a chemoattractant in the lower chamber (30 μL). T cells that migrated into the lower chambers after 3 h at 37°C were recovered and placed in standard Nunc 96‐well plates containing 70 μL of medium. The cells were fixed with an equal volume of 8% paraformaldehyde containing 2 μg mL^−1^ cell‐permeable Hoechst 33342. The cell number was enumerated using the IN Cell 1000 analyzer using 4× magnification. A total of 7 fields per well were imaged, resulting in ~85% of well coverage.

### Live cell migration on ICAM‐1/fc

Primary human T cells were re‐suspended at a density of 2 × 10^5^ cells mL^−1^ in 0.5% BSA/RPMI‐1640 and incubated for 2 h at 37°C. The cells were then incubated in 96‐well plates previously coated with 1 μg mL^−1^ ICAM‐1/Fc and 100 ng mL^−1^ SDF‐1α. Live cell brightfield imaging was subsequently performed at 37°C with the IN Cell 1000 analyzer and images were acquired every 50 s for 1 h. Automated cell tracking analysis was performed using ImagePro Plus 6.1 (Media Cybernetics, Rockville, USA). Three tracking parameters were analyzed: velocity, origin of distance and speed. The velocity of an object is defined as directionality (d)/time (t). The origin of distance is defined as the distance (D) from the starting point to the selected object. The speed is defined as the distance between tracking points/time (ImagePro Plus 6.0 for Windows Reference Guide, MediaCybernetics).

### ICAM‐1 adhesion assays

Adhesion of primary human T cells to ICAM‐1 was carried out using a static adhesion assay as described^55^ with minor modifications. In brief, the wells of a 96‐well plate were first coated with 5 μg mL^−1^ goat‐anti‐human IgG (Fc specific) in PBS and incubated overnight at 4°C. After removing unbound antibody and washing the wells with sterile PBS, ICAM‐1/Fc was added (1 μg mL^−1^ in sterile PBS) and incubated for 1 h at 37°C. Unbound ICAM‐1/Fc was removed and the wells were washed with sterile PBS. Primary human T cells that were deprived of IL‐2 and growth factors as described above were incubated in the wells at a density of 1 × 10^5^ cells mL^−1^ in the absence or presence of 100 ng mL^−1^ SDF‐1α and incubated for 30 min at 37°C. The wells were then gently filled with PBS and sealed with an adhesive 96‐well plate cover. The plates were inverted and centrifuged for 15 s at 6 *g* (the acceleration and brake on the centrifuge were set to 4, maximum setting = 9). Unbound cells were removed by keeping the plate inverted and gently flicking the liquid out of the wells. The plates were then filled with 4% paraformaldehyde containing 2 μg mL^−1^ cell‐permeable Hoechst 33342 and incubated overnight. The cell number was enumerated using the IN Cell 1000 analyzer using 4× magnification. A total of 7 fields per well were imaged.

### Confocal microscopy

Cells were seeded on 8‐well Lab‐tek coverslip chambers or glass‐bottomed 96‐well plates previously coated with poly L‐lysine or ICAM‐1/Fc (3 μg mL^−1^). After 2 h, the cells were fixed with 4% paraformaldehyde, permeabilized with 0.1% Triton‐X100/PBS and blocked with 5% BSA/PBS. The chambers/wells were subsequently incubated with anti‐α tubulin for 1 h at room temperature, washed three times with PBS and then incubated with AlexaFluor 488 goat‐anti‐mouse IgG for 30 min. After three washes in PBS, the cells were counterstained with phalloidin‐TRITC and Hoechst 33258. The slides were washed again and imaged using a Zeiss LSM510 Meta Duo Scan confocal microscope using a Plan‐Apochromat 63×/1.4 Oil DIC, 63× objective.

### Real‐time PCR assays

Real‐time PCR was used to quantify target gene knockdown of selected hits from the RNAi library screens. Total RNA was isolated from HuT 78 cells using the NucleoSpin RNA II kit (Macherey‐Nagel, Duren, Germany) 72 h post‐electroporation. A real‐time PCR reaction was performed using the QuantiTect primers and SYBR Green I dye following the manufacturers' instructions (Qiagen, Hilden, Germany) and analysis was performed on an ABI PRISM 7900 sequence detection system. Target gene reactions were normalized using QuantiTect GAPDH primers and the fold change expression was determined using the 2^−ΔΔCt^ method.^56^

### Western blotting

The cells were pelleted and lysed in ice‐cold 1% NP40 buffer containing 20 mM Tris–HCl (pH 7.5), 150 mM NaCl, 10% v/v glycerol, 5 mM sodium fluoride, 5 mM sodium pyrophosphate, 5 mM EDTA, 5 mM EGTA, 1 mM Na_3_VO_4_ and freshly added protease inhibitors (1 mM PMSF, 10 mg mL^−1^ leupeptin). Insoluble material was removed by centrifugation (12 000 *g* for 4 min at 4°C). The protein concentration was quantified by BCA assay. Equal concentrations of proteins were mixed with SDS‐PAGE sample buffer and heated to 100°C for 4 min. The samples were resolved on 10% SDS‐PAGE gels, transferred to PVDF membranes and blocked for 1 h at room temperature in 5% dried milk powder diluted in TBS‐0.1% Tween. After three washes in TBS‐0.1% Tween, the membranes were incubated overnight at 4°C with primary antibodies. After three washes in TBS‐0.1% Tween, the membranes were incubated with HRP‐labeled secondary antibodies for 1 h at room temperature. After three washes in TBS‐0.1% Tween, specific bands were visualized by incubating the membrane with ECL solution and exposing the membranes to light‐sensitive film. Densitometry was used to estimate the intensity of the bands using Kodak 1D Image Analysis Software (New Haven, USA).

### LFA‐1 affinity status

The affinity status of LFA‐1 was evaluated with the reporter antibodies KIM127 (extended conformation of β2 integrin) and mAb24 (high affinity β2 integrin). T cells (2 × 10^5^) were resuspended in 1% BSA/PBS followed by the addition of antibodies on ice for 30 min. The cells were washed in 1% BSA/PBS, incubated with AlexaFluor 488 goat anti‐mouse IgG for 10 min, washed again and analyzed by flow cytometry (Cyan ADP). The mean fluorescence intensity was quantified using the Cyan ADP analysis software and was expressed as a percentage relative to the control siRNA sample which was set at 100%.

### Statistical analysis

Statistical analysis was performed using the test outlined in the relevant Figure captions. Effects were deemed to be significant at *P*‐values < 0.05.

## AUTHOR CONTRIBUTIONS

**Michael Freeley:** Conceptualization; data curation; formal analysis; investigation; methodology; writing – original draft; writing – review and editing. **Antje Haap‐Hoff:** Conceptualization; data curation; formal analysis; investigation; methodology; validation. **Eugene Dempsey:** Data curation; formal analysis; investigation; methodology. **Dara Dunican:** Data curation; formal analysis; investigation; methodology. **Emily Bennett:** Data curation; formal analysis; investigation; methodology; validation. **Denise Triglia:** Data curation; formal analysis; investigation; methodology; validation. **Joanna Skubis‐Zegadlo:** Data curation; formal analysis; investigation; methodology; validation. **Anthony Mitchell Davies:** Data curation; formal analysis; investigation; methodology; resources. **Dermot Kelleher:** Conceptualization; formal analysis; funding acquisition; investigation; project administration; resources; supervision; writing – review and editing. **Aideen Long:** Conceptualization; data curation; formal analysis; funding acquisition; investigation; methodology; project administration; resources; supervision; writing – review and editing.

## CONFLICT OF INTEREST

The authors declare no conflicts of interest.

## Supporting information


Supplementary figure 1
Supplementary figure 2

## Data Availability

The data that support the findings of this study are available from the corresponding author upon reasonable request.

## References

[1] Masopust D , Schenkel JM . The integration of T cell migration, differentiation and function. Nat Rev Immunol 2013; 13: 309–320. 23598650 10.1038/nri3442

[2] Comerford I , Kara EE , McKenzie DR , McColl SR . Advances in understanding the pathogenesis of autoimmune disorders: focus on chemokines and lymphocyte trafficking. Br J Haematol 2014; 164: 329–341. 24164387 10.1111/bjh.12616

[3] Alon R , Shulman Z . Chemokine triggered integrin activation and actin remodeling events guiding lymphocyte migration across vascular barriers. Exp Cell Res 2011; 317: 632–641. 21376176 10.1016/j.yexcr.2010.12.007

[4] Hogg N , Patzak I , Willenbrock F . The insider's guide to leukocyte integrin signalling and function. Nat Rev Immunol 2011; 11: 416–426. 21597477 10.1038/nri2986

[5] Serrador JM , Nieto M , Sánchez‐Madrid F . Cytoskeletal rearrangement during migration and activation of T lymphocytes. Trends Cell Biol 1999; 9: 228–233. 10354569 10.1016/s0962-8924(99)01553-6

[6] Volkov Y , Long A , McGrath S , Ni Eidhin D , Kelleher D . Crucial importance of PKC‐β(I) in LFA‐1‐mediated locomotion of activated T cells. Nat Immunol 2001; 2: 508–514. 11376337 10.1038/88700

[7] Soede RD , Wijnands YM , Van Kouteren‐Cobzaru I , Roos E . ZAP‐70 tyrosine kinase is required for LFA‐1‐dependent T cell migration. J Cell Biol 1998; 142: 1371–1379. 9732296 10.1083/jcb.142.5.1371 PMC2149357

[8] Evans R , Lellouch AC , Svensson L , McDowall A , Hogg N . The integrin LFA‐1 signals through ZAP‐70 to regulate expression of high‐affinity LFA‐1 on T lymphocytes. Blood 2011; 117: 3331–3342. 21200022 10.1182/blood-2010-06-289140

[9] Morin NA , Oakes PW , Hyun YM , *et al*. Nonmuscle myosin heavy chain IIA mediates integrin LFA‐1 de‐adhesion during T lymphocyte migration. J Exp Med 2008; 205: 195–205. 18195072 10.1084/jem.20071543 PMC2234359

[10] Xu X , Jaeger ER , Wang X , *et al*. Mst1 directs myosin IIa partitioning of low and higher affinity integrins during T cell migration. PLoS One 2014; 9: e105561. 25133611 10.1371/journal.pone.0105561 PMC4136924

[11] Feng C , Li YF , Yau YH , *et al*. Kindlin‐3 mediates integrin αLβ2 outside‐in signaling, and it interacts with scaffold protein receptor for activated‐C kinase 1 (RACK1). J Biol Chem 2012; 287: 10714–10726. 22334666 10.1074/jbc.M111.299594 PMC3322817

[12] Watts JK , Corey DR . Silencing disease genes in the laboratory and the clinic. J Pathol 2012; 226: 365–379. 22069063 10.1002/path.2993 PMC3916955

[13] Mohr SE , Perrimon N . RNAi screening: new approaches, understandings, and organisms. Wiley Interdiscip Rev RNA 2012; 3: 145–158. 21953743 10.1002/wrna.110 PMC3249004

[14] Mohr S , Bakal C , Perrimon N . Genomic screening with RNAi: results and challenges. Annu Rev Biochem 2010; 79: 37–64. 20367032 10.1146/annurev-biochem-060408-092949 PMC3564595

[15] Simpson KJ , Davis GM , Boag PR . Comparative high‐throughput RNAi screening methodologies in *C. elegans* and mammalian cells. New Biotechnol 2012; 29: 459–470. 10.1016/j.nbt.2012.01.003 22306616

[16] Heigwer F , Port F , Boutros M . RNA interference (RNAi) screening in *Drosophila* . Genetics 2018; 208: 853–874. 29487145 10.1534/genetics.117.300077 PMC5844339

[17] Evans L , Sailem H , Vargas PP , Bakal C . Inferring signalling networks from images. J Microsc 2013; 252: 1–7. 23841886 10.1111/jmi.12062 PMC4217379

[18] Collinet C , Stöter M , Bradshaw CR , *et al*. Systems survey of endocytosis by multiparametric image analysis. Nature 2010; 464: 243–249. 20190736 10.1038/nature08779

[19] Freeley M , Bakos G , Davies A , Kelleher D , Long A , Dunican DJ . A high‐content analysis toolbox permits dissection of diverse signaling pathways for T lymphocyte polarization. J Biomol Screen 2010; 15: 541–555. 20460253 10.1177/1087057110369703

[20] Freeley M , Long A . Advances in siRNA delivery to T‐cells: potential clinical applications for inflammatory disease, cancer and infection. Biochem J 2013; 455: 133–1347. 24070422 10.1042/BJ20130950

[21] Legate KR , Wickström SA , Fässler R . Genetic and cell biological analysis of integrin outside‐in signaling. Genes Dev 2009; 23: 397–418. 19240129 10.1101/gad.1758709

[22] Mace EM , Monkley SJ , Critchley DR , Takei F . A dual role for talin in NK cell cytotoxicity: activation of LFA‐1‐mediated cell adhesion and polarization of NK cells. J Immunol 2009; 182: 948–956. 19124737 10.4049/jimmunol.182.2.948

[23] Katagiri K , Imamura M , Kinashi T . Spatiotemporal regulation of the kinase Mst1 by binding protein RAPL is critical for lymphocyte polarity and adhesion. Nat Immunol 2006; 7: 919–928. 16892067 10.1038/ni1374

[24] Katagiri K , Katakai T , Ebisuno Y , Ueda Y , Okada T , Kinashi T . Mst1 controls lymphocyte trafficking and interstitial motility within lymph nodes. EMBO J 2009; 28: 1319–1331. 19339990 10.1038/emboj.2009.82 PMC2683056

[25] Smith A , Bracke M , Leitinger B , Porter JC , Hogg N . LFA‐1‐induced T cell migration on ICAM‐1 involves regulation of MLCK‐mediated attachment and ROCK‐dependent detachment. J Cell Sci 2003; 116: 3123–3133. 12799414 10.1242/jcs.00606

[26] Heasman SJ , Carlin LM , Cox S , Ng T , Ridley AJ . Coordinated RhoA signaling at the leading edge and uropod is required for T cell transendothelial migration. J Cell Biol 2010; 190: 553–563. 20733052 10.1083/jcb.201002067 PMC2928012

[27] Belkina NV , Liu Y , Hao JJ , Karasuyama H , Shaw S . LOK is a major ERM kinase in resting lymphocytes and regulates cytoskeletal rearrangement through ERM phosphorylation. Proc Natl Acad Sci USA 2009; 106: 4707–4712. 19255442 10.1073/pnas.0805963106 PMC2660762

[28] Fong AM , Premont RT , Richardson RM , Yu YRA , Lefkowitz RJ , Patel DD . Defective lymphocyte chemotaxis in beta‐arrestin2‐ and GRK6‐deficient mice. Proc Natl Acad Sci USA 2002; 99: 7478–7483. 12032308 10.1073/pnas.112198299 PMC124256

[29] Herroeder S , Reichardt P , Sassmann A , *et al*. Guanine nucleotide‐binding proteins of the G12 family shape immune functions by controlling CD4^+^ T cell adhesiveness and motility. Immunity 2009; 30: 708–720. 19409815 10.1016/j.immuni.2009.02.010

[30] Block H , Stadtmann A , Riad D , *et al*. Gnb isoforms control a signaling pathway comprising Rac1, Plcβ2, and Plcβ3 leading to LFA‐1 activation and neutrophil arrest *in vivo* . Blood 2016; 127: 314–324. 26468229 10.1182/blood-2015-06-651034 PMC4722285

[31] Gibson SA , Benveniste EN . Protein kinase CK2: an emerging regulator of immunity. Trends Immunol 2018; 39: 82–85. 29307449 10.1016/j.it.2017.12.002 PMC5800982

[32] Ulges A , Witsch EJ , Pramanik G , *et al*. Protein kinase CK2 governs the molecular decision between encephalitogenic T_H_ 17 cell and T_reg_ cell development. Proc Natl Acad Sci USA 2016; 113: 10145–10150. 27555590 10.1073/pnas.1523869113 PMC5018788

[33] Gibson SA , Yang W , Yan Z , *et al*. Protein kinase CK2 controls the fate between Th17 cell and regulatory T cell differentiation. J Immunol 2017; 198: 4244–4254. 28468969 10.4049/jimmunol.1601912 PMC5512439

[34] Jang SW , Hwang SS , Kim HS , *et al*. Casein Kinase 2 is a critical determinant of the balance of Th17 and Treg cell differentiation. Exp Mol Med 2017; 49: e375. 28883547 10.1038/emm.2017.132 PMC5628272

[35] Yang W , Gibson SA , Yan Z , *et al*. Protein kinase 2 (CK2) controls CD4^+^ T cell effector function in the pathogenesis of colitis. Mucosal Immunol 2020; 13: 788–798. 31988467 10.1038/s41385-020-0258-x PMC7382987

[36] Dong G , Yang Y , Zhang H , *et al*. Protein kinase CK2 maintains reciprocal balance between Th17 and Treg cells in the pathogenesis of UC. Inflamm Bowel Dis 2022; 28: 830–842. 34904630 10.1093/ibd/izab312

[37] Bouvard D , Millon‐Fremillon A , Dupe‐Manet S , Block MR , Albiges‐Rizo C . Unraveling ICAP‐1 function: toward a new direction? Eur J Cell Biol 2006; 85: 275–282. 16546571 10.1016/j.ejcb.2005.10.005

[38] Freeley M , O'Dowd F , Paul T , *et al*. L‐plastin regulates polarization and migration in chemokine‐stimulated human T lymphocytes. J Immunol 2012; 188: 6357–6370. 22581862 10.4049/jimmunol.1103242

[39] Schaffner‐Reckinger E , Machado RAC . The actin‐bundling protein L‐plastin – a double‐edged sword: beneficial for the immune response, maleficent in cancer. Int Rev Cell Mol Biol 2020; 355: 109–154. 32859369 10.1016/bs.ircmb.2020.05.004

[40] Krummel MF , Bartumeus F , Gérard A . T cell migration, search strategies and mechanisms. Nat Rev Immunol 2016; 16: 193–201. 26852928 10.1038/nri.2015.16 PMC4869523

[41] Shen B , Delaney MK , Du X . Inside‐out, outside‐in, and inside‐outside‐in: G protein signaling in integrin‐mediated cell adhesion, spreading, and retraction. Curr Opin Cell Biol 2012; 24: 600–606. 22980731 10.1016/j.ceb.2012.08.011 PMC3479359

[42] Chang DD , Wong C , Smith H , Liu J . ICAP‐1, a novel beta1 integrin cytoplasmic domain‐associated protein, binds to a conserved and functionally important NPXY sequence motif of beta1 integrin. J Cell Biol 1997; 138: 1149–1157. 9281591 10.1083/jcb.138.5.1149 PMC2136751

[43] Köchl R , Thelen F , Vanes L , *et al*. WNK1 kinase balances T cell adhesion versus migration *in vivo* . Nat Immunol 2016; 17: 1075–1083. 27400149 10.1038/ni.3495 PMC4994873

[44] Kendirli A , de la Rosa C , Lämmle KF , *et al*. Identification of essential modules regulating T cell migration to the central nervous system in multiple sclerosis. Nat Neurosci 2023; 26: 1713–1725. 37709997 10.1038/s41593-023-01432-2 PMC10545543

[45] Rogers LM , Wang Z , Mott SL , Dupuy AJ , Weiner GJ . A genetic screen to identify gain‐ and loss‐of‐function modifications that enhance T‐cell infiltration into tumors. Cancer Immunol Res 2020; 8: 1206–1214. 32611665 10.1158/2326-6066.CIR-20-0056 PMC7483799

[46] Johansen KH , Golec DP , Huang B , *et al*. A CRISPR screen targeting PI3K effectors identifies RASA3 as a negative regulator of LFA‐1–mediated adhesion in T cells. Sci Signal 2022; 15: eabl9169. 35857633 10.1126/scisignal.abl9169 PMC9637254

[47] Bartelt RR , Cruz‐Orcutt N , Collins M , Houtman JCD . Comparison of T cell receptor‐induced proximal signaling and downstream functions in immortalized and primary T cells. PLoS One 2009; 4: e5430. 19412549 10.1371/journal.pone.0005430 PMC2673025

[48] Lefort CT , Kim M . Human T lymphocyte isolation, culture and analysis of migration *in vitro* . J Vis Exp 2010; 40: 2017. 10.3791/2017 PMC3153890 20526279

[49] Freeley M , Long A . The two hit hypothesis: an improved method for siRNA‐mediated gene silencing in stimulated primary human T cells. J Immunol Methods 2013; 396: 116–127. 23988722 10.1016/j.jim.2013.08.005

[50] Birmingham A , Selfors LM , Forster T , *et al*. Statistical methods for analysis of high‐throughput RNA interference screens. Nat Methods 2009; 6: 569–575. 19644458 10.1038/nmeth.1351 PMC2789971

[51] Kozak K , Bakos G , Hoff A , *et al*. Workflow‐based software environment for large‐scale biological experiments. J Biomol Screen 2010; 15: 892–899. 20625182 10.1177/1087057110377354

[52] Bar‐Joseph Z , Demaine ED , Gifford DK , Srebro N , Hamel AM , Jaakkola TS . K‐ary clustering with optimal leaf ordering for gene expression data. Bioinformatics 2003; 19: 1070–1078. 12801867 10.1093/bioinformatics/btg030

[53] Cline MS , Smoot M , Cerami E , *et al*. Integration of biological networks and gene expression data using cytoscape. Nat Protoc 2007; 2: 2366–2382. 17947979 10.1038/nprot.2007.324 PMC3685583

[54] Bindea G , Mlecnik B , Hackl H , *et al*. ClueGO: a cytoscape plug‐in to decipher functionally grouped gene ontology and pathway annotation networks. Bioinformatics 2009; 25: 1091–1093. 19237447 10.1093/bioinformatics/btp101 PMC2666812

[55] Konstandin MH , Wabnitz GH , Aksoy H , Kirchgessner H , Dengler TJ , Samstag Y . A sensitive assay for the quantification of integrin‐mediated adhesiveness of human stem cells and leukocyte subpopulations in whole blood. J Immunol Methods 2007; 327: 30–39. 17719602 10.1016/j.jim.2007.07.005

[56] Livak KJ , Schmittgen TD . Analysis of relative gene expression data using real‐time quantitative PCR and the 2−ΔΔCT method. Methods 2001; 25: 402–408. 11846609 10.1006/meth.2001.1262

