# Supplementary material for: RNAi library screening reveals Gβ1, Casein Kinase 2 and ICAP‐1 as novel regulators of LFA‐1‐mediated T cell polarity and migration
Source: Immunol Cell Biol. 2024 Nov 28;103(1):73–92. doi: 10.1111/imcb.12838 (PMC11688611; doi:10.1111/imcb.12838)
Supplement: Supplementary file 1 — Supplementary figure 1 Supplementary figure 2 [file IMCB-103-73-s001.pdf]

Gene	Gene symbol	Approved Name	1-Form Factor	Nuclear Displacement	Cell Area	Cell Gyration Radius	Cell Elongation Factor
3611	ILK	integrin-linked kinase	-2.59	-1.95	-2.68	-2.84	-2.32
26659	OR7A5	olfactory receptor, family 7, subfamily A, member 5	-2.64	-2.48	-2.58	-2.71	-1.55
6789	STK4	serine/threonine kinase 4	-3.01	-2.76	-2.55	-2.65	-3.01
2870	GRK6	G protein-coupled receptor kinase 6	-2.26	-2.13	-1.82	-2.07	-2.41
23538	OR52A1	olfactory receptor, family 52, subfamily A, member 1	-0.64	-2.11	-2.30	-2.20	-2.25
4159	MC3R	melanocortin 3 receptor	-2.90	-0.91	-3.08	-2.34	-1.98
2850	GPR27	G protein-coupled receptor 27	-3.08	-3.02	0.17	-2.80	-3.04
5585	PRKCL1	protein kinase N1	-0.95	-3.43	-0.62	-2.17	-2.91
23566	EDG7	lysophosphatidic acid receptor 3	-0.95	-2.24	-0.57	-2.00	-1.79
5347	PLK1	polo-like kinase 1	-0.91	-0.06	-0.71	-3.22	-6.88
143503	MGC24137	olfactory receptor, family 51, subfamily E, member 1	-1.14	-1.78	-1.02	-1.34	-1.91
90	ACVR1	activin A receptor, type I	-0.69	-2.03	-0.77	-1.40	-1.62
5128	PCTK2	cyclin-dependent kinase 17	0.18	-2.10	-0.55	-0.61	-2.14
80045	GPR157	G protein-coupled receptor 157	-0.42	-1.88	-0.45	-0.14	-2.13
7371	UMPK	uridine-cytidine kinase 2	-0.29	-2.08	-0.15	-0.03	-1.95
23049	SMG1	smg-1 homolog, phosphatidylinositol 3-kinase-related kinase (C. elegans)	0.57	-2.28	0.27	-0.38	-1.97
51347	JIK	TAO kinase 3	-0.45	-1.83	0.38	-0.77	-2.28
2011	MARK2	MAP/microtubule affinity-regulating kinase 2	-0.26	-2.03	0.48	-0.83	-3.00
29775	CARD10	caspase recruitment domain family, member 10	-1.74	-0.49	-0.56	-0.94	-1.83
269	AMHR2	anti-Mullerian hormone receptor, type II	-0.38	-1.06	-0.80	-0.98	-1.82
60385	TSKS	testis-specific serine kinase substrate	0.33	-0.24	-0.56	0.07	-3.23
55577	NAGK	N-acetylglucosamine kinase	0.26	0.01	-0.54	-0.70	-1.59
5218	PFTK1	cyclin-dependent kinase 14	-0.53	-0.56	-0.40	-0.38	-2.21
701	BUB1B	budding uninhibited by benzimidazoles 1 homolog beta (yeast)	-0.79	-0.68	-0.34	-0.88	-2.11
5286	PIK3C2A	phosphoinositide-3-kinase, class 2, alpha polypeptide	-0.09	-0.51	-0.16	-0.86	-2.22
1158	CKM	creatine kinase, muscle	-0.10	-0.63	-0.12	-0.48	-2.15
6010	RHO	rhodopsin	-0.19	-0.14	0.09	0.89	-2.39
1848	DUSP6	dual specificity phosphatase 6	-0.10	-0.73	0.18	-0.49	-1.65
54963	URKL1	uridine-cytidine kinase 1-like 1	0.56	-0.38	0.43	0.08	-2.15
552	AVPR1A	arginine vasopressin receptor 1A	0.41	-0.86	0.45	-0.27	-1.79
7272	TTK	TTK protein kinase	0.57	-1.23	0.60	-0.34	-2.03
5531	PPP4C	protein phosphatase 4, catalytic subunit	1.10	-0.48	1.12	0.79	-2.05
147	ADRA1B	adrenergic, alpha-1B-, receptor	-2.77	-2.08	-2.53	-2.76	-0.53
1455	CSNK1G2	casein kinase 1, gamma 2	-2.71	-1.80	-2.21	-2.10	-0.48
2782	GNB1	guanine nucleotide binding protein (G protein), beta polypeptide 1	-3.17	-0.69	-2.68	-2.15	-0.11
1263	PLK3	polo-like kinase 3	-2.69	-0.15	-2.68	-2.93	0.31
8986	RPS6KA4	ribosomal protein S6 kinase, 90kDa, polypeptide 4	-3.17	-0.34	-2.02	-1.77	-0.13
5211	PFKL	phosphofructokinase, liver	-1.88	-0.78	-1.74	-1.83	0.07
51755	CRK7	cyclin-dependent kinase 12	-0.92	-0.62	-2.61	-2.07	0.04
259285	TAS2R39	taste receptor, type 2, member 39	-0.51	-0.51	-2.27	-1.86	0.08
1452	CSNK1A1	casein kinase 1, alpha 1	-1.31	-0.05	-2.16	-2.45	-0.36
5163	PDK1	pyruvate dehydrogenase kinase, isozyme 1	-0.60	-0.84	-1.68	-1.70	0.20
6787	NEK4	NIMA (never in mitosis gene a)-related kinase 4	-0.39	-0.73	-1.63	-1.71	-0.98
11318	ADMR	G protein-coupled receptor 182	-1.95	-2.31	-0.35	-1.76	-0.13
165082	GPR113	G protein-coupled receptor 113	-0.73	-1.53	-1.31	-2.07	-0.65
135	ADORA2A	adenosine A2a receptor	-1.23	-1.65	-1.09	-1.72	-0.53
9448	MAP4K4	mitogen-activated protein kinase kinase kinase kinase 4	-0.02	-2.02	-1.06	-1.94	-0.81
197258	FUK	fucokinase	-1.73	-0.83	-1.34	-1.61	-0.57
7433	VIPR1	vasoactive intestinal peptide receptor 1	-2.17	-1.29	-1.10	-1.55	-0.16
6915	TBXA2R	thromboxane A2 receptor	-2.12	-0.92	-0.95	-1.58	-0.50
2785	GNG3	guanine nucleotide binding protein (G protein), gamma 3	-2.17	-1.09	-0.64	-1.69	0.12
4140	MARK3	MAP/microtubule affinity-regulating kinase 3	-0.74	-1.06	-0.98	-1.83	-0.40
2915	GRM5	glutamate receptor, metabotropic 5	-0.92	-1.05	-0.75	-1.74	-1.15
23432	GPR161	G protein-coupled receptor 161	-2.23	-0.74	-2.66	-1.16	-0.06
8590	OR6A2	olfactory receptor, family 6, subfamily A, member 2	-0.74	-0.48	-6.14	-0.96	-0.42
10087	COL4A3BP	collagen, type IV, alpha 3 (Goodpasture antigen) binding protein	0.34	-0.70	-2.38	-0.30	-0.12
10149	GPR64	G protein-coupled receptor 64	-0.66	0.60	-2.00	0.39	0.42
11245	GPR	G protein-coupled receptor 1	-0.83	-0.77	-1.95	-1.08	0.26
23596	OPN3	opsin 3	0.04	-0.31	-1.82	-0.69	-0.65
6387	CXCL12	chemokine (C-X-C motif) ligand 12	-0.84	-0.81	-1.81	-1.04	-0.61
1880	EBI2	G protein-coupled receptor 183	-0.60	-0.80	-1.80	-0.81	-0.79
6850	SYK	spleen tyrosine kinase	-0.96	-0.33	-1.71	-0.99	0.27
10886	GPR74	neuropeptide FF receptor 2	-1.22	-0.60	-1.70	-1.31	-0.19
23683	PRKCN	protein kinase D3	-0.34	-0.23	-1.59	-0.91	-0.16
23266	LPHN2	latrophilin 2	-2.53	-1.96	-1.11	-1.26	-0.51
816	CAMK2B	calcium/calmodulin-dependent protein kinase II beta	-1.84	-2.16	-0.92	-0.95	-0.77
154	ADRB2	adrenergic, beta-2-, receptor, surface	-0.84	-1.70	-0.99	-1.01	-0.64
7273	TTN	titin	-0.39	-1.62	-0.63	-0.83	-0.55
8550	MAPKAPK5	mitogen-activated protein kinase-activated protein kinase 5	-0.46	-1.74	-0.51	-0.76	-0.66
7201	TRHR	thyrotropin-releasing hormone receptor	-0.71	-2.07	-0.51	-1.16	-1.15
5616	PRKY	protein kinase, Y-linked, pseudogene	-0.21	-2.24	-0.33	-0.51	-0.90
26353	HSPB8	heat shock 22kDa protein 8	0.28	-1.53	-0.33	-0.75	-0.66
65266	PRKWNK4	WNK lysine deficient protein kinase 4	-0.48	-2.40	-0.19	-0.88	-0.84
2769	GNA15	guanine nucleotide binding protein (G protein), alpha 15 (Gq class)	-2.19	-1.15	-1.43	-1.45	-0.49
8208	CHAF1B	chromatin assembly factor 1, subunit B (p60)	-1.95	0.35	-1.27	-0.54	2.08
6300	MAPK12	mitogen-activated protein kinase 12	-1.99	0.53	-1.14	-0.95	0.22
5032	P2RY11	purinergic receptor P2Y, G-protein coupled, 11	-2.15	-1.02	-1.07	-0.99	-0.44
53831	GPR84	G protein-coupled receptor 84	-2.36	0.20	-0.86	-0.28	0.96
9020	MAP3K14	mitogen-activated protein kinase kinase kinase 14	-2.25	-0.62	-0.82	-0.78	0.04
1399	CRKL	v-crk sarcoma virus CT10 oncogene homolog (avian)-like	-1.93	-1.18	-0.79	-1.11	0.05
11035	RIPK3	receptor-interacting serine-threonine kinase 3	-2.46	0.12	-0.72	-0.55	0.13
64080	RBKS	ribokinase	-1.70	-0.59	-0.70	-0.73	0.02
5030	P2RY4	pyrimidinergic receptor P2Y, G-protein coupled, 4	-3.25	-0.92	-0.68	-0.66	0.49
7432	VIP	vasoactive intestinal peptide	-2.33	-0.51	-0.60	-0.56	-1.16
4881	NPR1	natriuretic peptide receptor A/guanylate cyclase A (atrionatriuretic peptide receptor A)	-2.06	-0.65	-0.56	-0.60	0.94
2261	FGFR3	fibroblast growth factor receptor 3	-1.56	-0.17	-0.54	-0.80	0.08
51265	CDKL3	cyclin-dependent kinase-like 3	-1.70	-0.89	-0.53	-0.40	0.31
317705	VN1R5	vomeronasal 1 receptor 5 (gene/pseudogene)	-1.93	-0.64	-0.47	-0.73	-0.41
23729	CARKL	sedoheptulokinase	-2.25	-0.46	-0.33	-0.36	0.44
91754	NEK9	NIMA (never in mitosis gene a)- related kinase 9	-2.19	0.79	-0.22	0.25	2.69
2853	GPR31	G protein-coupled receptor 31	-1.91	-0.25	-0.18	-0.42	0.35
3274	HRH2	histamine receptor H2	-1.66	-0.01	0.04	-0.15	-0.23
57147	PACE-1	SCY1-like 3 (S. cerevisiae)	-1.86	0.39	0.07	-0.57	0.27
10333	TLR6	toll-like receptor 6	-1.78	-0.06	0.13	-1.11	0.15
3717	JAK2	Janus kinase 2	-5.02	-0.20	0.32	-0.07	-0.19
2859	GPR35	G protein-coupled receptor 35	-1.94	-0.30	0.92	0.54	1.17

Gene	Gene symbol	Approved Name	1-Form Factor	Nuclear Displacement	Cell Area	Cell Gyration Radius	Cell Elongation Factor
6093	ROCK1	Rho-associated, coiled-coil containing protein kinase 1	4.96	6.19	1.87	3.33	2.04
9475	ROCK2	Rho-associated, coiled-coil containing protein kinase 2	4.17	4.64	3.18	4.32	2.69
139760	GPR119	G protein-coupled receptor 119	0.14	2.15	0.25	1.55	2.50
1460	CSNK2B	casein kinase 2, beta polypeptide	0.95	2.56	0.40	2.16	2.01
1741	DLG3	discs, large homolog 3 (Drosophila)	0.74	1.97	0.48	1.65	1.57
2693	GHSR	growth hormone secretagogue receptor	0.48	2.76	0.55	2.08	2.47
1457	CSNK2A1	casein kinase 2, alpha 1 polypeptide	1.37	1.94	0.72	1.66	1.93
56670	GPR91	succinate receptor 1	0.39	2.95	0.96	2.08	1.84
6416	MAP2K4	mitogen-activated protein kinase kinase 4	-0.01	2.18	1.00	1.75	1.91
317702	VN1R3	vomeronasal 1 receptor 3 (gene/pseudogene)	0.72	2.65	1.03	2.52	2.83
139760	GPR119	G protein-coupled receptor 119	-0.11	1.76	-0.68	0.36	2.38
5894	RAF1	v-raf-1 murine leukemia viral oncogene homolog 1	0.25	2.30	-0.51	-0.16	2.13
6872	TAF1	TAF1 RNA polymerase II, TATA box binding protein (TBP)-associated factor, 250kDa	0.70	3.86	-0.41	1.09	3.69
5832	PYCS	aldehyde dehydrogenase 18 family, member A1	-0.19	2.45	-0.19	0.17	2.23
2788	GNG7	guanine nucleotide binding protein (G protein), gamma 7	0.44	2.29	0.27	0.46	2.25
2534	FYN	FYN oncogene related to SRC, FGR, YES	0.28	1.94	0.33	1.03	1.79
11069	RAPGEF4	Rap guanine nucleotide exchange factor (GEF) 4	-0.26	2.00	0.42	0.81	2.10
84636	FKSG79	G protein-coupled receptor 174	0.70	1.73	0.78	1.01	1.85
79646	PANK3	pantothenate kinase 3	-0.09	0.73	-0.84	-0.12	1.76
83394	PITPNM3	PITPNM family member 3	-0.74	0.32	-0.52	-0.09	1.68
6753	SSTR3	somatostatin receptor 3	-0.27	-0.08	-0.44	-0.80	2.60
916	CD3E	CD3e molecule, epsilon (CD3-TCR complex)	0.75	0.52	-0.31	0.09	2.55
10755	RGS19IP1	GIPC PDZ domain containing family, member 1	-0.42	0.49	-0.28	0.10	1.90
1269	CNR2	cannabinoid receptor 2 (macrophage)	0.05	1.00	-0.27	0.59	2.15
8834	C17ORF35	transmembrane protein 11	-0.08	0.63	-0.26	0.45	2.00
208	AKT2	v-akt murine thymoma viral oncogene homolog 2	-0.37	1.26	-0.18	-0.22	1.91
5681	PSKH1	protein serine kinase H1	-0.50	0.20	-0.11	0.13	1.94
1326	MAP3K8	mitogen-activated protein kinase kinase kinase 8	-0.54	0.86	0.19	0.24	2.12
79541	OR2A4	olfactory receptor, family 2, subfamily A, member 4	0.13	0.46	0.21	0.95	1.96
8392	OR3A3	olfactory receptor, family 3, subfamily A, member 3	-0.26	0.73	0.58	0.03	2.51
3269	HRH1	histamine receptor H1	0.65	1.12	0.65	1.12	1.63
3716	JAK1	Janus kinase 1	0.57	1.70	1.91	2.10	0.78
6733	SRPK2	SRSF protein kinase 2	0.09	1.97	1.99	1.66	0.97
5598	MAPK7	mitogen-activated protein kinase 7	0.23	2.31	2.45	2.21	0.00
84059	MASS1	G protein-coupled receptor 98	0.87	1.74	2.52	2.35	0.82
5739	PTGIR	prostaglandin I2 (prostacyclin) receptor (IP)	0.39	2.86	2.57	3.62	-0.20
6794	STK11	serine/threonine kinase 11	1.70	0.76	1.75	1.68	0.04
6002	RGS12	regulator of G-protein signaling 12	2.05	1.07	1.93	2.02	0.57
985	CDC2L2	cyclin-dependent kinase 11A	2.98	0.84	1.95	1.87	0.51
29953	TRHDE	thyrotropin-releasing hormone degrading enzyme	2.33	0.47	2.18	2.00	0.46
91807	LOC91807	myosin light chain kinase 3	2.00	0.76	2.39	2.19	0.11
553	AVPR1B	arginine vasopressin receptor 1B	2.31	0.86	2.40	2.35	0.87
10672	GNA13	guanine nucleotide binding protein (G protein), alpha 13	3.74	-0.10	3.70	3.43	-0.92
94	ACVRL1	activin A receptor type II-like 1	-0.59	0.76	1.70	1.57	0.07
149420	LOC149420	PDLIM1 interacting kinase 1 like	0.70	0.93	1.75	2.16	0.50
5313	PKLR	pyruvate kinase, liver and RBC	0.84	0.88	1.82	2.12	-0.46
1019	CDK4	cyclin-dependent kinase 4	0.80	0.69	2.98	2.56	0.34
23620	NTSR2	neurotensin receptor 2	2.17	2.39	0.42	1.67	0.33
5062	PAK2	p21 protein (Cdc42/Rac)-activated kinase 2	0.63	1.98	0.87	1.81	0.89
5879	RAC1	ras-related C3 botulinum toxin substrate 1 (rho family, small GTP binding protein Rac1)	0.80	0.48	-0.04	2.72	1.22
317701	VN1R2	vomeronasal 1 receptor 2	-0.31	0.26	0.15	1.77	0.66
2185	PTK2B	PTK2B protein tyrosine kinase 2 beta	0.52	-0.16	0.29	1.82	0.82
7083	TK1	thymidine kinase 1, soluble	0.26	-0.03	0.31	2.14	0.31
148	ADRA1A	adrenergic, alpha-1A-, receptor	0.63	0.16	0.40	1.76	0.59
9942	XYLB	xylulokinase homolog (H. influenzae)	0.67	1.01	0.81	1.95	0.34
4216	MAP3K4	mitogen-activated protein kinase kinase kinase 4	-0.20	2.50	2.18	1.19	0.80
8386	OR1D5	olfactory receptor, family 1, subfamily D, member 5	1.69	0.58	1.56	0.31	0.14
6793	STK10	serine/threonine kinase 10	1.70	0.51	1.65	1.04	0.21
2535	FZD2	frizzled family receptor 2	2.25	0.48	1.88	0.84	-0.09
369	ARAF1	v-raf murine sarcoma 3611 viral oncogene homolog	1.69	0.49	2.16	1.36	0.09
4882	NPR2	natriuretic peptide receptor B/guanylate cyclase B (atrionatriuretic peptide receptor B)	0.06	1.13	1.60	1.07	0.48
4354	MPP1	membrane protein, palmitoylated 1, 55kDa	0.98	0.45	1.68	0.83	-0.03
1233	CCR4	chemokine (C-C motif) receptor 4	0.63	-0.08	1.75	0.63	-0.26
9019	MPZL1	myelin protein zero-like 1	0.87	0.47	1.84	0.90	-0.64
2872	MKNK2	MAP kinase interacting serine/threonine kinase 2	-0.05	0.76	2.25	1.11	0.42
256710	MGC26856	GLI pathogenesis-related 1 like 1	0.19	0.16	2.28	0.29	-0.52
8325	FZD8	frizzled family receptor 8	1.22	0.15	2.56	0.74	-0.68
5631	PRPS1	phosphoribosyl pyrophosphate synthetase 1	0.53	0.62	3.63	0.55	-0.15
9263	STK17A	serine/threonine kinase 17a	2.10	2.27	0.01	0.59	0.77
9270	ITGB1BP1	integrin beta 1 binding protein 1	2.03	2.65	0.05	0.53	0.58
57410	SCYL1	SCY1-like 1 (S. cerevisiae)	0.24	2.19	-0.95	-0.48	0.42
5105	PCK1	phosphoenolpyruvate carboxykinase 1 (soluble)	-0.10	2.09	-0.23	-0.04	0.99
29110	TBK1	TANK-binding kinase 1	0.11	1.88	-0.20	0.20	0.77
2868	GRK4	G protein-coupled receptor kinase 4	0.73	2.26	-0.20	0.80	1.32
6361	CCL17	chemokine (C-C motif) ligand 17	-0.10	2.87	-0.14	-0.34	-0.93
6885	MAP3K7	mitogen-activated protein kinase kinase kinase 7	-1.09	1.56	-0.12	0.37	0.94
1432	MAPK14	mitogen-activated protein kinase 14	-0.14	1.97	-0.07	0.64	0.85
6046	BRD2	bromodomain containing 2	0.39	1.93	0.11	0.09	0.41
2866	GPR42	G protein-coupled receptor 42 (gene/pseudogene)	0.51	1.82	0.17	0.59	0.63
8390	OR1G1	olfactory receptor, family 1, subfamily G, member 1	-0.08	2.64	0.21	-0.28	0.08
10256	CNKSR1	connector enhancer of kinase suppressor of Ras 1	-0.74	6.03	0.34	0.75	-0.23
6348	CCL3	chemokine (C-C motif) ligand 3	0.75	2.06	0.42	1.22	1.08
5607	MAP2K5	mitogen-activated protein kinase kinase 5	-0.28	2.52	0.49	0.87	0.34
64123	ELTD1	EGF, latrophilin and seven transmembrane domain containing 1	0.88	1.71	0.68	1.18	1.26
5595	MAPK3	mitogen-activated protein kinase 3	-0.10	1.58	0.70	0.89	0.50
1119	CHKA	choline kinase alpha	0.79	3.34	0.74	1.06	1.08
29993	PACSIN1	protein kinase C and casein kinase substrate in neurons 1	0.21	1.96	0.81	0.81	0.43
9815	GIT2	G protein-coupled receptor kinase interacting ArfGAP 2	0.30	1.66	0.86	1.03	1.04
9344	TAO1	TAO kinase 2	0.63	2.56	0.89	1.22	0.88
4294	MAP3K10	mitogen-activated protein kinase kinase kinase 10	1.12	1.84	0.90	1.17	0.33
7535	ZAP70	zeta-chain (TCR) associated protein kinase 70kDa	-0.24	2.90	0.92	0.67	0.42
80835	TAS1R1	taste receptor, type 1, member 1	0.78	1.64	0.98	1.18	0.95
4883	NPR3	natriuretic peptide receptor C/guanylate cyclase C (atrionatriuretic peptide receptor C)	1.70	-0.62	-0.50	-0.30	-0.59
57396	CLK4	CDC-like kinase 4	2.50	0.03	-0.04	0.09	0.00
5515	PPP2CA	protein phosphatase 2, catalytic subunit, alpha isozyme	2.65	-0.06	0.03	0.21	-0.18
920	CD4	CD4 molecule	1.61	-0.34	0.04	0.10	0.41
2596	GAP43	growth associated protein 43	1.70	-0.82	0.07	0.00	-0.02
6000	RGS7	regulator of G-protein signaling 7	1.63	0.25	0.26	0.61	-0.07
1111	CHEK1	checkpoint kinase 1	2.30	0.88	0.32	0.36	0.13
338398	TAS2R60	taste receptor, type 2, member 60	1.63	0.22	0.34	0.21	0.03
3362	HTR6	5-hydroxytryptamine (serotonin) receptor 6, G protein-coupled	1.92	0.46	0.38	0.73	-0.38
51765	MST4	Serine/threonine-protein kinase MST4	2.84	-0.21	0.38	-0.35	-0.27
205	AK3	adenylate kinase 3	2.32	0.15	0.39	0.36	-0.32
1608	DGKG	diacylglycerol kinase, gamma 90kDa	1.60	0.96	0.51	1.06	0.23
4882	NPR2	natriuretic peptide receptor B/guanylate cyclase B (atrionatriuretic peptide receptor B)	1.70	0.56	0.55	0.85	0.43
4638	MYLK	myosin light chain kinase	2.47	0.86	0.58	0.71	0.41
206426	MGC26597	phosphatidylinositol-4-phosphate 5-kinase, type I, pseudogene 1	2.11	-0.01	0.60	0.59	0.47
140901	STK35	serine/threonine kinase 35	1.58	0.72	0.64	1.03	0.46
2844	GPR21	G protein-coupled receptor 21	1.72	1.06	0.70	0.77	0.24
846	CASR	calcium-sensing receptor	1.75	0.05	0.76	0.37	0.10
1022	CDK7	cyclin-dependent kinase 7	1.64	0.19	0.80	0.61	-0.01
8566	PDXK	pyridoxal (pyridoxine, vitamin B6) kinase	1.86	0.82	0.84	0.89	-0.42
140469	MYO3B	myosin IIIB	2.32	0.35	0.85	0.57	-0.71
8601	RGS20	regulator of G-protein signaling 20	2.23	-0.72	1.05	0.58	-0.13
2826	GPR2	chemokine (C-C motif) receptor 10	2.25	0.25	1.16	0.88	-0.27

Decrease | Unchanged | Increase

Supplementary figure 1

Supplementary figure 1. RNAi library screen hits and corresponding effects on cell morphology parameters is shown for both kinome RNAi library and the GPCR-GPCR-associated RNAi library. The hits from the library screens were grouped together according to the number of morphology parameters that were perturbed as a result of siRNA knockdown. The black arrows indicate the hits referred to in the main text and Figure 2.

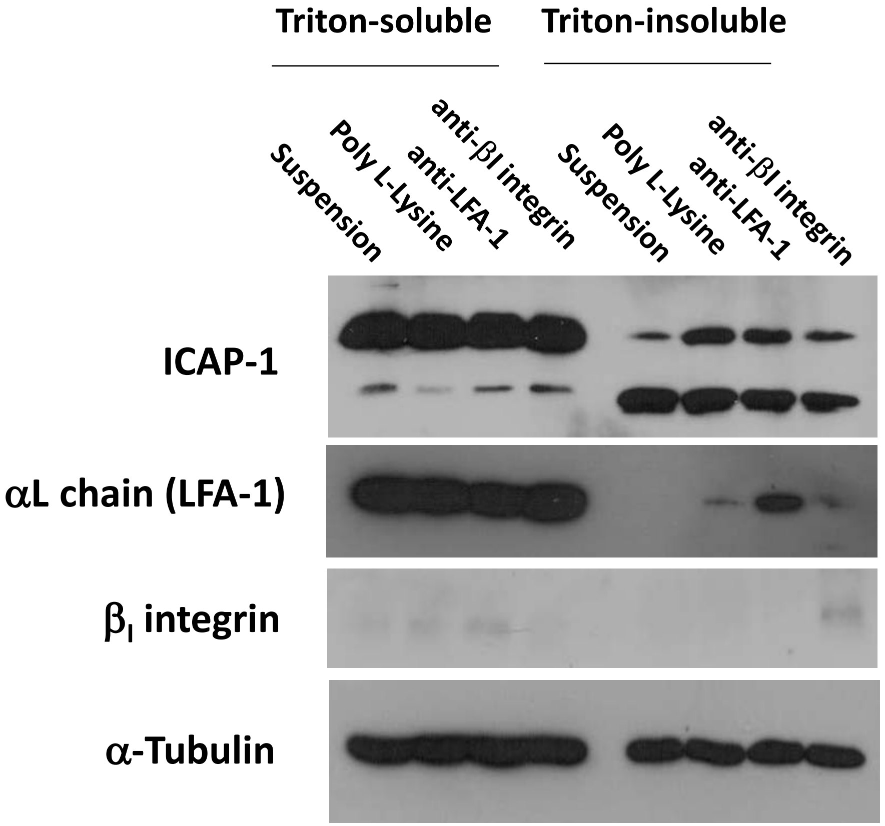

Supplementary figure 2. ICAP-1 associates with β1 and β2 integrin complexes in primary human T cells. Primary human T cells were incubated on anti-LFA-1 or anti-β1 integrin antibodies to induce a polarised phenotype, or as a control incubated on poly-L-lysine (adhesion control) or left in suspension. The cells were treated with a Triton X100 detergent-based buffer to extract soluble proteins while insoluble cytoskeletal-associated proteins remain attached to the plate. Soluble and insoluble fractions were resolved by SDS-PAGE and probed by western blotting for the indicated proteins.

Supplementary figure 2
